# Avid binding by B cells to the *Plasmodium* circumsporozoite protein repeat suppresses responses to protective subdominant epitopes

**DOI:** 10.1016/j.celrep.2021.108996

**Published:** 2021-04-13

**Authors:** Deepyan Chatterjee, Fiona J. Lewis, Henry J. Sutton, Joe A. Kaczmarski, Xin Gao, Yeping Cai, Hayley A. McNamara, Colin J. Jackson, Ian A. Cockburn

**Affiliations:** 1 Department of Immunology and Infectious Disease, John Curtin School of Medical Research, The Australian National University, Canberra, ACT 2601, Australia; 2 Research School of Chemistry, The Australian National University, Canberra, ACT 2601, Australia

**Keywords:** immunodominance, B cells, malaria, *Plasmodium falciparum*, malaria vaccines, circumsporozoite protein, immunodomination

## Abstract

Antibodies targeting the NANP/NVDP repeat domain of the *Plasmodium falciparum* circumsporozoite protein (CSP_Repeat_) can protect against malaria. However, it has also been suggested that the CSP_Repeat_ is a decoy that prevents the immune system from mounting responses against other domains of CSP. Here, we show that, following parasite immunization, B cell responses to the CSP_Repeat_ are immunodominant over responses to other CSP domains despite the presence of similar numbers of naive B cells able to bind these regions. We find that this immunodominance is driven by avid binding of the CSP_Repeat_ to cognate B cells that are able to expand at the expense of B cells with other specificities. We further show that mice immunized with repeat-truncated CSP molecules develop responses to subdominant epitopes and are protected against malaria. These data demonstrate that the CSP_Repeat_ functions as a decoy, but truncated CSP molecules may be an approach for malaria vaccination.

## Highlights

• The repeat domain is immunodominant within the circumsporozoite protein
• High avidity responses by repeat-specific B cells inhibit subdominant responses
• The number of naive B cell precursors does not predict immunodominance hierarchies
• Vaccination with repeat-truncated circumsporozoite proteins induces robust protection

## Graphical abstract

**Figure:**
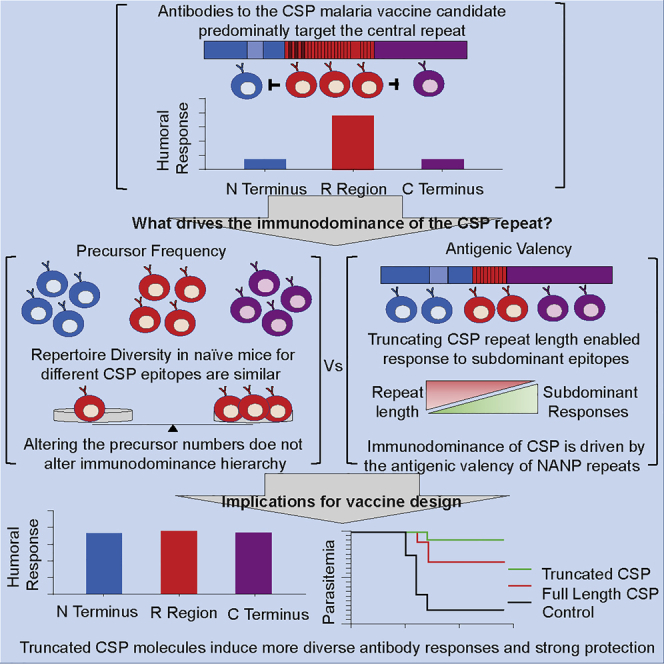

## Teaser

Chatterjee et al. show that avid B cell responses to repeating epitopes can suppress B cell responses to other regions of the same protein, driving immunodominance hierarchies. In the context of malaria vaccination, circumsporozoite-based immunogens carrying truncated repeat regions stimulated more diverse antibody responses and induced robust protection.

## Introduction

The most advanced malaria vaccine RTS,S/AS01 aims to induce antibodies that target the repeat region of the circumsporozoite protein (CSP), which covers the surface of the *Plasmodium* sporozoite (Agnandji et al., 2012; Olotu et al., 2016; RTS,S Clinical Trials Partnership, 2015). The rationale for this approach derives from the observation that immunization with radiation-attenuated sporozoites confers sterile protection against malaria and that the humoral response induced by irradiated sporozoites is dominated by anti-CSP antibodies (Ishizuka et al., 2016; Nussenzweig et al., 1967; Seder et al., 2013; Zavala et al., 1985). Early studies demonstrated that monoclonal antibodies (mAbs) targeting the repeat regions of the *P. berghei* CSP molecule protected mice against challenge with the rodent parasite *Plasmodium berghei* (Ferreira et al., 1987; Potocnjak et al., 1980; Yoshida et al., 1980). More recently, human mAbs targeting the NANP/NVDP repeat domain of the *Plasmodium falciparum* circumsporozoite protein (CSP_Repeat_) have also been shown to be protective in preclinical mouse models (Kisalu et al., 2018; Tan et al., 2018; Triller et al., 2017).

Despite the demonstrated protective capacity of CSP_Repeat_-specific Abs, it has also been argued that the CSP_Repeat_ is an immunodominant “decoy” that distracts the immune system from making protective responses against other epitopes within CSP or other proteins on the sporozoite surface (Schofield, 1990; Schofield and Uadia, 1990). Evidence for the immunodominance of the CSP_Repeat_ initially came from early studies that showed that a short (NANP)_3_ peptide based on this domain could absorb most sporozoite binding activity of sera from hyperimmune individuals (Zavala et al., 1985). In support of the concept that the responses to CSP_Repeat_ are sub-optimal, large amounts of anti-CSP_Repeat_ mAbs are required for protection in preclinical challenge models, whereas in RTS,S clinical trials, protection requires very high amounts (>50 μg/mL) of anti-CSP_Repeat_ antibody (Kisalu et al., 2018; Tan et al., 2018; Triller et al., 2017; White et al., 2015). In contrast, antibody responses to other regions of CSP are less well understood. One small epidemiological study associated increased levels of antibodies targeting the N terminal domain of CSP (CSP_Nterm_) with protection from clinical disease (Bongfen et al., 2009). Subsequently, a mouse mAb, 5D5, targeting an epitope within the CSP_Nterm_ was found to be protective against sporozoite challenge (Espinosa et al., 2015). More recently, human mAbs targeting the junction between the CSP_Nterm_ and CSP_Repeat_ were found to be protective (Kisalu et al., 2018; Tan et al., 2018). Antibodies targeting the C-terminal domain CSP (CSP_Cterm_) have been associated with protection by the RTS,S vaccine in clinical trials (Dobaño et al., 2019; Ubillos et al., 2018), although individual mAbs targeting this domain have not been found to confer protection (Triller et al., 2017).

Beyond *Plasmodium*, there has been increased interest in the factors that drive B cell immunodominance and how they can be manipulated for improved vaccination outcomes. Recent findings in influenza and HIV immunology have revealed the existence of broadly neutralizing antibodies (bnAbs); however, they target rare subdominant epitopes. In HIV, it was recently shown that transferred B cells carrying a germline version of the bnAbs VRC01 or 3BNC60 could be induced to compete successfully in germinal centers (GCs) if the number of naive precursors was artificially increased or after immunization with polyvalent immunogens that bound the B cells with greater avidity (Abbott et al., 2018; Dosenovic et al., 2018; Kato et al., 2020). However, these studies focused on the response to a single antigen and did not investigate the effect of avidity or precursor number on the hierarchy of competing immune responses to different epitopes within an antigen or pathogen. This question has been partially addressed for influenza for which it has been shown that broadly neutralizing responses to the stem regions of hemagglutinin (HA) can be favored over responses to the immunodominant—but highly variable—head region by immunization with stem-only constructs, even if delivered alongside full-length HA (Angeletti et al., 2019).

Given the highlighted roles for antigen poly-valency and precursor numbers in driving B cell immunodominance, we investigated whether these factors drive the CSP_Repeat_ to be immunodominant. Moreover, we wanted to know whether the response to the repeat was itself inhibiting responses to other antigens within CSP. The finding that CSP_Repeat_ can be bound avidly by B cells from a range of immunoglobulin gene families suggested that there may be high numbers of precursors for this domain (Fisher et al., 2017; Kisalu et al., 2018; Murugan et al., 2018; Tan et al., 2018). More suggestively, we and others have shown that the CSP_Repeat_ can be bound by 6 or more specific antibodies (Fisher et al., 2017; Kisalu et al., 2018; Oyen et al., 2017, 2018), and it has been demonstrated that the repeat can crosslink multiple B cell receptors (BCRs) to enhance B cell signaling (Imkeller et al., 2018). Accordingly, we tested the roles of these factors in driving the dominance of the antibody response against the CSP_Repeat_ and determined if we could manipulate the immunodominance hierarchy to develop better vaccination protocols.

## Results

### The circumsporozoite protein repeat domain is immunodominant

**Figure 1:**
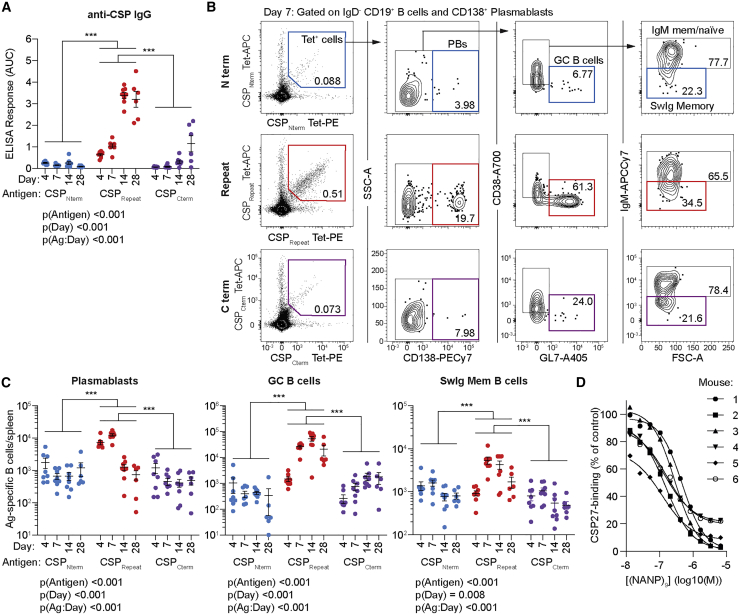
Responses to the CSP_Repeat_ are immunodominant over responses to other domains C57BL/6 mice were immunized with 5 × 10^4^ irradiated Pb-PfSPZ sporozoites; blood and spleens were taken at 4, 7, 14, and 28 days post-immunization for analysis by ELISA and flow cytometry by using probes specific for each domain of CSP (CSP_Cterm_, CSP_Repeat_, and CSP_Nterm_). (A) IgG responses to each domain measured by ELISA; data are shown as area under the curve. (B) Representative flow cytometry plots from a single mouse at the day 7 time point showing the gating of PBs, GC B cells, and SwIg Mem for antigen-specific IgD^−^ B cells identified using tetramers specific for the CSP_Cterm_, CSP_Repeat_, and CSP_Nterm_; values are percentages. (C) Absolute numbers of (i) plasmablasts, (ii) GC B cells, and (iii) SwIg memory cells in each mouse for each antigen (domain). (D) IgG binding to CSP27 of day 28 sera from each Pb-PfSPZ immunized mouse after incubation with different concentrations of CSP_Repeat_ peptide measured by ELISA. Data for (A) and (C) are represented as mean ± SD pooled from two independent experiments (n = 3–5 mice/time point/experiment); data were analyzed via two-way ANOVA, with experiment and mouse included in the model as fixed factors; ANOVA p values are listed below or adjacent to each graph. Pairwise comparisons were performed using a Tukey post-test, and significant pairwise comparisons are represented as symbols; ^∗^p < 0.05, ^∗∗^p < 0.01, ^∗∗∗^p < 0.001.

To formally test the immunodominance of responses to the CSP_Repeat_ over responses to the CSP_Nterm_ and CSP_Cterm_, we immunized mice with irradiated *P. berghei* parasites engineered to express *P. falciparum* CSP in place of the endogenous *P. berghei* CSP molecule (Pb-PfSPZ), which carry a full-length (4NVDP/38NANP) *P. falciparum* CSP gene (Figures S1A and S1B; Espinosa et al., 2017). At days 4, 7, 14, and 28 post-immunization, sera were taken for antibody analysis by ELISA with domain-specific peptides, and spleens were taken for cellular analysis by flow cytometry. The structural fidelity of our peptides was verified using mAbs specific for the different domains of PfCSP (Figure S1C; Espinosa et al., 2015; Kisalu et al., 2018; Scally et al., 2018; Zavala et al., 1983). Immunoglobulin G (IgG) responses to the CSP_Repeat_ were significantly higher than responses to either of the other domains, with a significant response developing to the CSP_Cterm_ only after 28 days (Figure 1A).

We further used tetramer probes based on our domain-specific peptides to track the total numbers and phenotypes of B cells responding to each domain of CSP over time by using flow cytometry (Figure 1B; Figure S2A). The response to sporozoites is characterized by an early plasmablast (PB) response that wanes and leaves a prolonged GC reaction (Fisher et al., 2017). Four days after immunization of mice with Pb-PfSPZ, the number of CSP_Repeat_^+^ CD138^+^ PBs was ∼10-fold higher than the number of CSP_Nterm_^+^ or CSP_Cterm_^+^ PBs (Figure 1Ci). By day 7, a pronounced GC reaction developed and the number of CSP_Repeat_^+^ GL7^+^ B cells was again ∼10-fold higher than responses to the other domains, which was sustained until day 28 (Figure 1Cii). We further analyzed the number of IgD^−^ IgM^−^ (switched Ig; SwIg) CD38^+^ memory (Mem) B cells and found that the immunodominance of the response to the CSP_Repeat_ extended into the memory phase of the immune response (Figure 1Ciii).

We were concerned that our peptides might have different sensitivities to detect antibodies and B cells specific for the different domains, and this may explain the differences in the responses we measured. Therefore, to further test the immunodominance of the repeat, we tested the ability of our repeat peptide to inhibit the binding of sporozoite immune sera to CSP27 (Cerami et al., 1992), a slightly truncated recombinant CSP molecule carrying 27 repeats (3 NVDPs and 24 NANPs; Figure S1B) described. We found that for 6/6 sera taken from mice 28 days after Pb-PfSPZ immunization, the repeat peptide was able to inhibit >70% of the binding to CSP27, indicating that the majority of anti-CSP-specific antibodies induced by sporozoite immunization target this region (Figure 1D).

### B cell precursor number does not predict the immunodominance of the repeat

Previous studies have highlighted the fact that increasing the number of precursors for an antigen led to a concomitant increase in the number of cells entering GCs; the same studies also highlighted roles for antigen valency in allowing responses to successfully compete in the GC (Abbott et al., 2018; Dosenovic et al., 2018). To determine if there was an association between the number of precursors and the immunodominance hierarchy, we calculated the number of precursors specific for each domain by using a dual-tetramer approach. Whole splenocytes from naïve mice were co-stained with a full-length CSP-phycoerythrin (PE) probe and a domain-specific probe conjugated to allophycocyanin (APC) prior to magnetic enrichment by using anti-PE microbeads. To exclude streptavidin-specific B cells that could bind to both our probes, cells were further co-stained with streptavidin-BV510 conjugated to an irrelevant peptide. The number of precursors per spleen were then quantified by flow cytometry (Figure S2B). As a control for non-specific binding, we also enriched splenocytes from MD4 mice that carry a transgenic BCR specific for hen egg lysozyme (HEL) and thus should not harbor CSP-specific B cells (Goodnow et al., 1989).

**Figure 2:**
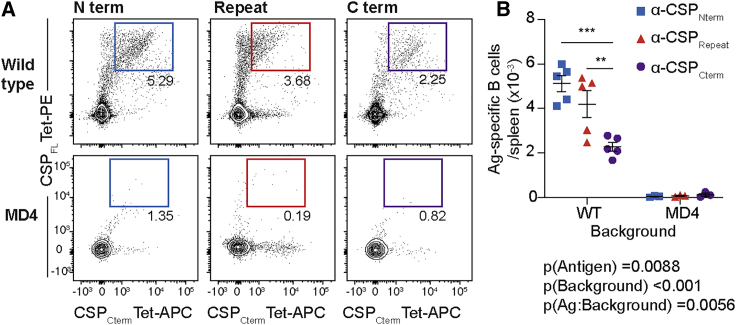
The number of antigen-specific precursors does not predict the immunodominance hierarchy within CSP The number of precursors for each domain of CSP in C57BL/6 and MD4 mice was quantified using full-length CSP conjugated to PE and domain-specific probes conjugated to APC. (A) Representative flow cytometry plots showing the staining of antigen-specific cells in each mouse background. (B) Summary data from (A), mean ± SEM shown, all data were analyzed via two-way ANOVA, and ANOVA p values are listed below or adjacent to each graph; data are pooled from two independent experiments (n = 2–3 mice/group/experiment). Pairwise comparisons were performed using a Tukey post-test ,and significant pairwise comparisons are represented as symbols; ^∗^p < 0.05, ^∗∗^p < 0.01, ^∗∗∗^p < 0.001.

Using a dual tetramer approach, we found that the number of repeat specific B cells in a naive spleen numbered around ∼4 × 10^3^, or a frequency of 1 in ∼6 × 10^3^ (Figures 2A and 2B). This finding was not significantly different from the number of CSP_Nterm_-specific B cells, although it was around 2-fold higher than the number of CSP_Cterm_-specific B cells. As expected, antigen-specific cells were rare in MD4 mice, confirming that our staining was specific. Interestingly the numbers of CSP_Nterm_ and CSP_Cterm_ precursors appear to exceed the number of cells responding to these antigens after immunization, suggesting that the response to these antigens may be strongly suppressed by the immunodominant response to the CSP_Repeat_. This is consistent with previous findings that the majority of antigen-specific precursors do not respond to immunization even in the absence of competition (Taylor et al., 2015). Overall, there was no strong relationship between the number of B cell precursors specific for each domain and the magnitude of the response to each domain after sporozoite immunization.

### Increasing anti-CSP precursor B cell number does not suppress responses to other antigens when precursors are abundant

Our finding that the number of precursors does not predict the immunodominance hierarchy contrasts with work that correlates precursor numbers with the magnitude of the ensuing response (Abbott et al., 2018; Dosenovic et al., 2018). However, previous studies did not examine how the response to one antigen might affect the responses to another linked antigen, a concept that could be described as immunodomination. Accordingly, we developed a system in which we could modulate both the number of precursors and the valency of two competing antigens in the context of CSP. We achieved this by conjugating the 4-hydroxy-3-nitrophenyl (NP) acetyl-hapten to recombinant CSP by crosslinking on lysine. Because lysine residues are not found in CSP_Repeat_, the NP-hapten would bind exclusively to the N- and C-terminal domains (Figure S3A). As proof of principle, we found that immunization with CSP27 conjugated to 2 NP moieties (CSP27-NP2) was able to induce strong anti-NP and anti-CSP_Repeat_ IgG responses (Figures S3B and S3C). We were then able to modulate the number of precursors for CSP_Repeat_-specific B cells by using Igh^g2A10^ B cells, which carry the germline heavy chain of the CSP_Repeat_-specific 2A10 antibody (McNamara et al., 2020). Similarly, we could modulate the number of NP-specific B cells by using the established B1-8^hi^ mouse system (Shih et al., 2002a, 2002b). Finally, we could alter the valency of the response to NP or the repeat by conjugating more NP molecules per CSP or by reducing the length of the CSP_Repeat_ domain.

**Figure 3:**
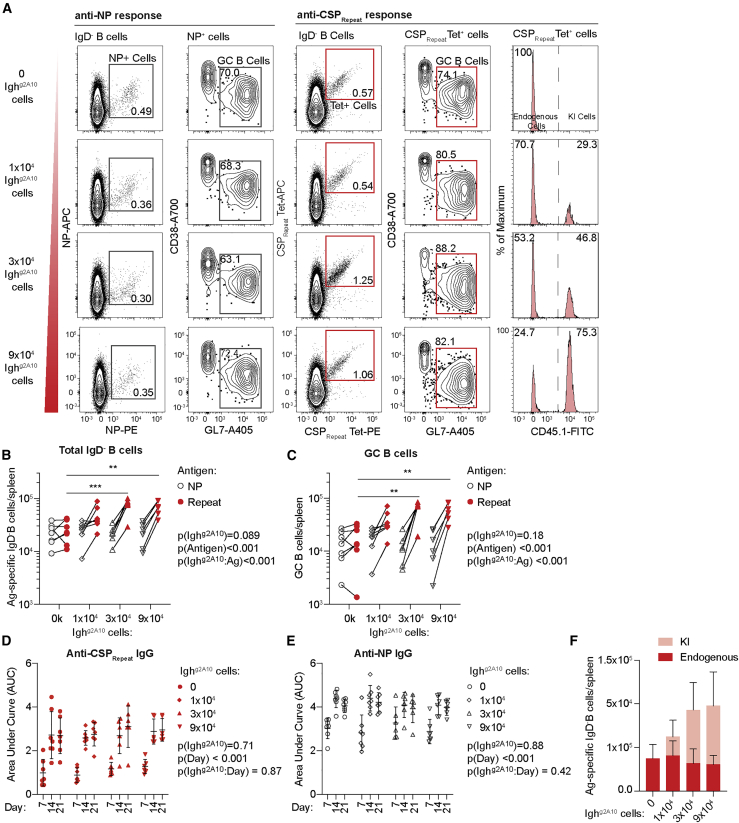
Increasing the CSP_Repeat_-specific precursor number does not suppress the endogenous response to linked antigens 0, 1 × 10^4^, 3 × 10^4^, or 9 × 10^4^ of CD45.1 Igh^g2A10^ cells were adoptively transferred into C57BL/6 mice followed by immunization with 30 μg CSP27-NP2 in alum. Sera were taken on days 7, 14, and 21 and spleens analyzed 21 days post-immunization. (A) Representative flow cytometry plots showing gating of total IgD^−^ and GC B cells specific for NP or the CSP_Repeat_; values are percentages. (B) Absolute numbers of NP probe^+^ and CSP_Repeat_ tetramer^+^ IgD^−^ B cells. (C) Absolute numbers of NP probe^+^ and CSP_Repeat_ tetramer^+^ GC B cells. (D) Total IgG response to CSP_Repeat_ measured by (NANP)_9_ ELISA. (E) Total IgG response to NP measured by NP(14)BSA ELISA. (F) Absolute numbers of CSP_Repeat_ tetramer^+^ CD45.1^+^ Igh^g2A10^ and CD45.1^−^ endogenous cells. Data are represented as mean ± SD pooled from two independent experiments (n ≥ 3 mice/group/experiment); all data were analyzed via two-way ANOVA, with experiment and mouse included in the model as fixed factors. ANOVA p values are listed below or adjacent to each graph. Pairwise comparisons were performed using a Tukey post-test, and significant values are represented as symbols; ^∗^p < 0.05, ^∗∗^p < 0.01, ^∗∗∗^p < 0.001.

To determine the role of precursor number in driving immunodominance, we adoptively transferred defined numbers of CSP_Repeat_ tetramer^+^, CD45.1 Igh^g2A10^ cells into CD45.2 C57BL/6 mice (Figure S2C). Mice were then immunized with CSP27-NP2; IgG responses were measured 7, 14, and 21 days post-immunization; and the number of NP and CSP_Repeat_-specific cells were quantified by flow cytometry on day 21 (Figure 3A). We hypothesized that increased anti-CSP_Repeat_ precursor number would not only increase the response to the CSP_Repeat_ but also suppress the NP immune response. As expected, increasing the number of CSP_Repeat_-specific precursors increased both the number of total CSP_Repeat_ binding B cells and CSP_Repeat_-specific GC B cells responding to this antigen (Figures 3B and 3C). Perhaps surprisingly, the magnitude of the antibody response was unaltered (Figure 3D). However, there was no concomitant decrease in the overall B cell and GC B cell response to NP (Figures 3B and 3C), and the magnitude of the IgG antibody response to NP was also unaffected by the addition of Igh^g2A10^ cells (Figure 3E). Finally, because we could distinguish our transferred cells from the endogenous response by the expression of CD45.1, we were able to determine that the endogenous response to CSP was not suppressed by the addition of an enhanced number of germline precursors specific for CSP (Figure 3F).

### Increasing anti-CSP precursor B cell number does not suppress responses to other antigens when precursors are limiting

It has been estimated that NP-specific cells are present at a high frequency (1 in 4 × 10^3^ or ∼6 × 10^3^/spleen) in C57BL/6 mice (Weisel et al., 2016). Thus, the number of precursors for both the CSP_Repeat_ and NP are high in C57BL/6 mice. We were concerned that in the previous experiments we could not test whether immunodomination occurs in situations in which the number of precursors for a subdominant antigen is more limiting; for example, the frequency of VRC01-like antibody precursors has been estimated to be as low as 1 in 1 × 10^6^. Accordingly, we designed an experiment in which groups of MD4 mice, which lack endogenous cells able to respond to NP or CSP_Repeat_, received a variable number (0, 1 × 10^3^, 5 × 10^3^, or 2.5 × 10^4^) of Igh^g2A10^ cells and a fixed number (1 × 10^3^) of B1-8^hi^ cells for precursor ratios of 0:1, 1:1, 5:1, or 25:1. The number of 1 × 10^3^ was chosen, as it has previously been shown to result in a similar precursor frequency to the number of VRC01 precursors in humans (Abbott et al., 2018), consistent with ∼5% engraftment as reported previously (Dosenovic et al., 2018; Taylor et al., 2015). The recipient mice were subsequently immunized with CSP27-NP2 and antibody and B cell responses analyzed by ELISA and flow cytometry. We hypothesized that if precursor numbers were important as higher numbers of Igh^g2A10^ cells were transferred, the response to NP by the B1-8^hi^ cells would diminish.

**Figure 4:**
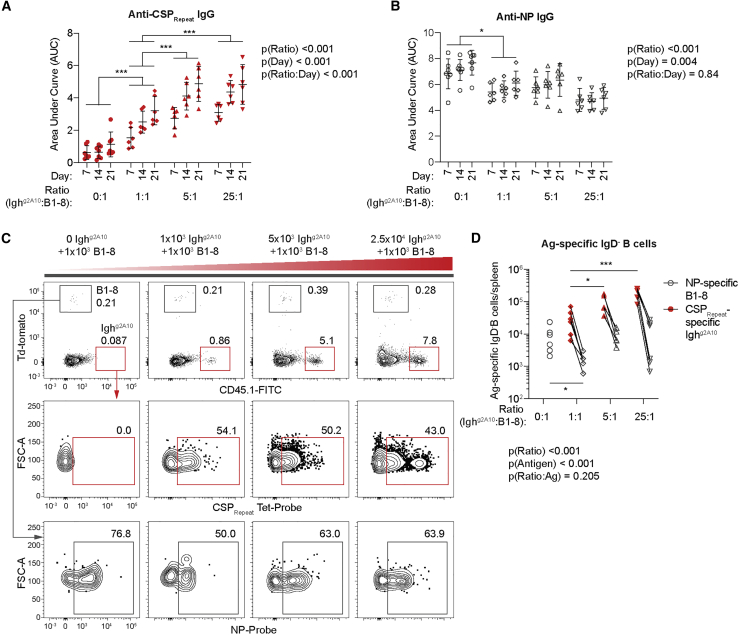
Increasing CSP_Repeat_-specific precursor number does not suppress the response by rare B cells specific for linked antigens 1 × 10^3^ B1-8^hi^ cells and 0, 1 × 10^3^, 5 × 10^3^, or 2.5 × 10^4^ CD45.1 Igh^g2A10^ cells were adoptively transferred into MD4 mice followed by immunization with 30 μg CSP27-NP2 in alum. Sera were taken on days 7, 14, and 21 and spleens analyzed 21 days post-immunization. (A) Total IgG response to CSP_Repeat_ measured by (NANP)_9_ ELISA. (B) Total IgG response to NP measured via NP(14)BSA ELISA. (C) Representative flow cytometry plots showing gating of total IgD^−^ and GC B cells specific for NP or the CSP_Repeat_; values are percentages. (D) Absolute numbers of NP probe^+^ and CSP_Repeat_ tetramer^+^ IgD^−^ B cells. Data are represented as mean ± SD pooled from two independent experiments (n ≥ 3 mice/group/experiment); all data were analyzed via two-way ANOVA, with experiment and mouse included in the model as fixed factors. ANOVA p values are listed below or adjacent to each graph. Pairwise comparisons to the 1:1 (control) group were performed using a Tukey post-test, and significant values are represented as symbols; ^∗^p < 0.05, ^∗∗^p < 0.01, ^∗∗∗^p < 0.001.

As expected, an increase in the number of Igh^g2A10^ cells led to an increase in the IgG response to the CSP_Repeat_, although this saturated if more than 5 × 10^3^ cells were transferred (Figure 4A). A strong anti-NP IgG response was detected in mice that received B1-8^hi^ cells but not Igh^g2A10^ cells, i.e., in the absence of any competition. This response was only marginally suppressed by the addition of 1 × 10^3^ Igh^g2A10^ cells (1:1 ratio), and further increases in the ratio of Igh^g2A10^ cells to B1-8^hi^ cells had no additional effect (Figure 4B). The antibody data were reflected at the cellular level, with a minimal suppressive effect on the response to CSP27-NP2 by B1-8^hi^ cells being observed as more Igh^g2A10^ cells were transferred and responded (Figures 4C and 4D). Collectively, our data in both wild-type and MD4 mice suggest that increasing the number of specific precursors for a given antigen increases the response to that antigen, but this increase has, at most, a minimal effect on parallel responses to other antigens.

### Reducing the valency of immunodominant antigens allows subdominant responses to expand

**Figure 5:**
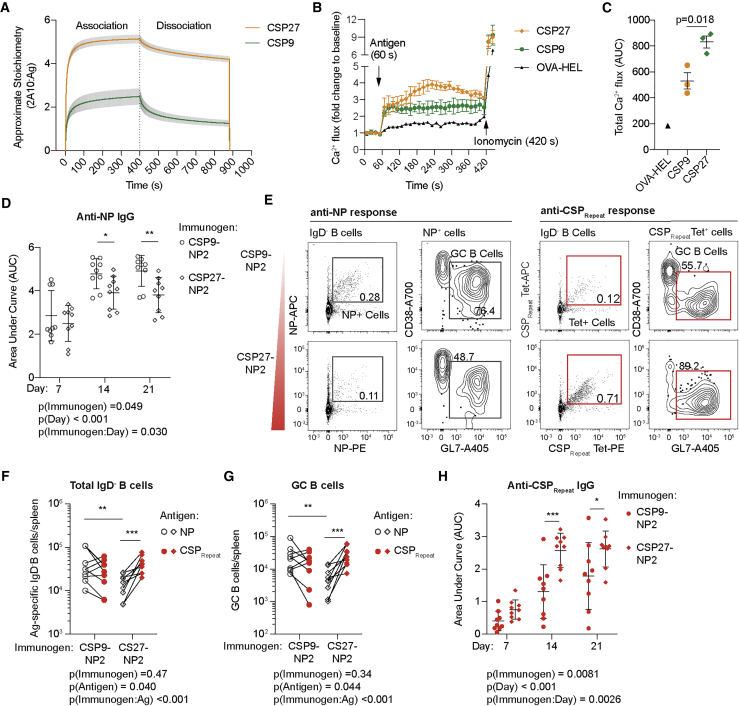
Decreasing the valency of the CSP_Repeat_ alters the immunodominance hierarchy Recombinant CSP9 was purified and conjugated to NP at a 1:2 ratio to generate CSP9-NP2, and mice were immunized with either CSP9-NP2 (23 μg) or CSP27-NP2 (30 μg) in alum; sera were taken on days 7, 14, and 21 and spleens analyzed 21 days post-immunization. (A) Approximate binding stoichiometry of the 2A10:Ag complex formed when a saturating concentration (2 μM) of mAb 2A10 was passed over immobilized CSP27 or CSP9; data show mean ± SD of two technical replicates (n = 2). (B) Calcium flux of sorted Igh^g2A10^ cells incubated with Indo-1 dye and stimulated with CSP27, CSP9, or OVA-HEL, and the Ca2^+^ flux was measured; near the end of the acquisition, ionomycin was added as a positive control; data show the mean ± SD of three experimental replicates with summary data. (C) Summary data from (B) analyzed via pairwise t test; mean ± SD shown. (D) Total IgG response to NP measured via NP(14)BSA ELISA. (E) Representative flow cytometry plots showing gating of total IgD^−^ and GC B cells specific for NP or the CSP_Repeat_; values are percentages. (F) Absolute numbers of NP probe^+^ and CSP_Repeat_ tetramer^+^ IgD^−^ B cells. (G) Absolute numbers of NP probe^+^ and CSP_Repeat_ tetramer^+^ GC B cells. (H) Total IgG response to CSP_Repeat_ measured via (NANP)_9_ ELISA. Data for (D)–(H) are represented as mean ± SD pooled from two independent experiments (n ≥ 4 mice/group/experiment); these data were analyzed via two-way ANOVA, with experiment and mouse included in the model as fixed factors. ANOVA p values are listed below or adjacent to each graph. Pairwise comparisons were performed using a Tukey post-test, and significant values are represented as symbols; ^∗^p < 0.05, ^∗∗^p < 0.01, ^∗∗∗^p < 0.001.

Given the repeating nature of the CSP_Repeat_, we next tested whether the ability of the long CSP_Repeat_ domain to crosslink multiple BCRs might drive the immunodominance of this domain. We further asked whether other responses were suppressed by the strong response to this multivalent antigen. Accordingly, we developed a construct that carried just nine NANP repeats (CSP9; Figure S1). Using a surface plasmon resonance saturation experiment, we found that CSP9 could only bind two to three 2A10 antibodies, compared to the five to six bound by CSP27 (Figure 5A), which is in line with previous structural and biophysical data from our laboratory (Fisher et al., 2017). Importantly, this reduced binding corresponded to a reduction in BCR signaling, as calcium fluxes were lower when Igh^g2A10^ cells were pulsed with CSP9 than those pulsed with CSP27 (Figures 5B and 5C).

To test whether the reduction in BCR signaling by CSP9 corresponded to a reduction in the immunodominance of the CSP_Repeat_, we compared responses to CSP27-NP2 and NP-haptenated CSP9 (CSP9-NP2). CSP9-NP2 had significantly elevated NP-specific IgG compared to NP2-CSP27, particularly on days 14 and 21 post-immunization (Figure 5D). An analysis of the cellular NP and CSP_Repeat_ responses by flow cytometry at day 21 revealed that truncation of the CSP_Repeat_ not only reduced the number of cells responding to the CSP_Repeat_ but also allowed for a significant increase in the number of NP-specific total B cells and GC cells supporting the increase in antibody titers (Figures 5E–5G). Moreover, the CSP_Repeat_ IgG titers were significantly decreased for NP2-CSP9 immunized mice compared to those of NP2-CSP27 (Figure 5H). Overall, these data support the importance of valency in determining immunodominance, as decreasing the repeat length shifted the response away from the CSP_Repeat_ and toward NP.

We next performed the converse experiment and compared responses to CSP27, CSP27-NP2, CSP27-NP6 ,and CSP27-NP10. Under these conditions, the length of the CSP_Repeat_ antigen is fixed but the valency of NP varies. In agreement with the previous finding, increasing the NP:CSP ratio not only increased the response to NP (Figure S4A) but also decreased the level of antibodies to the CSP_Repeat_ (Figure S4B). Increasing the NP:CSP ratio resulted in a switch in the immunodominance hierarchy; in mice immunized with CSP27-NP2, the CSP_Repeat_ response was immunodominant, whereas the NP response dominated the CSP_Repeat_ response upon immunization with CSP27-NP10 (Figures S4C–S4E). Collectively, these data indicate a powerful role for antigen valency in driving the immunodominance of repeating antigens.

### Immunization with repeat-truncated CSP molecules induces antibodies against subdominant epitopes and robust protection against live parasite challenge

**Figure 6:**
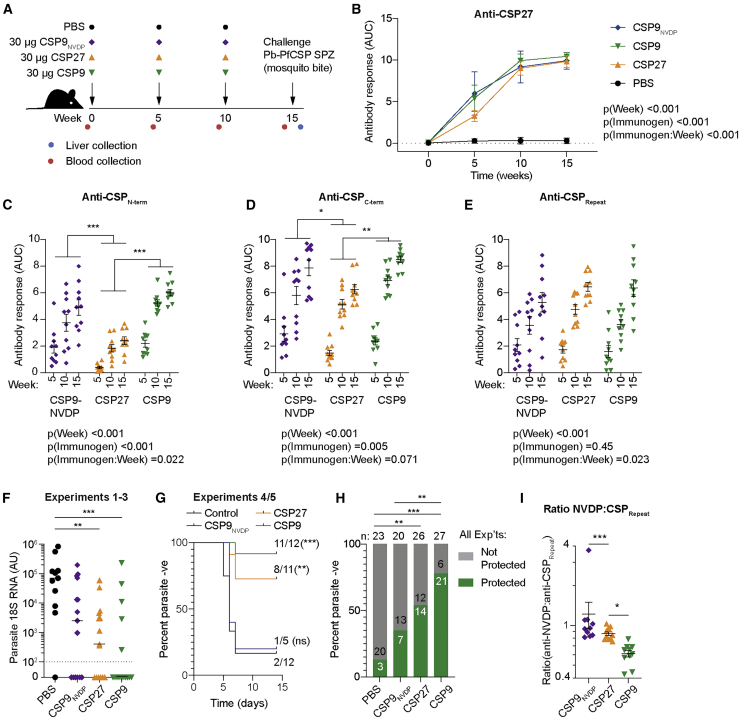
Immunization with truncated CSP molecules induces diverse antibody responses that protect against malaria C57BL/6 mice were immunized three times at 5-week intervals with 30 μg CSP9, CSP9_NVDP_, CSP27, or alum alone and challenged via mosquito bite with Pb-PfSPZ sporozoites; blood was drawn prior to each immunization and challenge for analysis of the antibody response. (A) Schematic of the experiment. (B) Overall IgG responses to CSP27. (C) Overall IgG responses to CSP_Nterm_. (D) Overall IgG responses to CSP_Cterm_. (E) Overall IgG responses to CSP_Repeat_. Data in (B)–(E) were from two experiments with five mice/experiment/group, analyzed via two-way ANOVA with experiment and mouse as blocking factors. ANOVA p values are listed below or adjacent to each graph; pairwise comparisons between groups (averaged over time) were performed using a Tukey post-test; and significant values are represented as symbols; ^∗^p < 0.05, ^∗∗^p < 0.01, ^∗∗∗^p < 0.001. (F) Parasite burden measured via 18S RNA in the livers of mice 42 h post challenge; data were pooled from three experiments with three to five mice/experiment/group and analyzed via Kruskal-Wallis test with Dunn’s multiple comparisons test. (G) The proportion of immunized mice that remain free of the blood stage parasite over 14 days post-infectious mosquito bite challenge; data were pooled from two experiments, and pairwise comparisons with the control group were analyzed via log-rank (Mantel-Cox) test. (H) Proportion of mice from both (F) and (G) (five experiments in total) with no detectable parasite RNA/or that remain sub-patent in each group; pairwise comparisons were made via Fisher’s exact test. ^∗^p < 0.05, ^∗∗^p < 0.01, ^∗∗∗^p < 0.001. (I) Ratio of the week 15 response to the NVDP peptide and the standard CSP_Repeat_ peptide between the different immunization groups; data were from two experiments with five mice/experiment/group analyzed via one-way ANOVA with experiment as a blocking factor.

One prediction of our data is that immunization with CSP9 will induce stronger responses to the CSP_Nterm_ and CSP_Cterm_ than CSP27. Moreover, if these non-CSP_Repeat_ responses have anti-parasitic effects, then immunization with CSP9 should be more protective than immunization with CSP27. Accordingly, we measured antibody responses in mice immunized three times at 5-week intervals with CSP9 and CSP27 formulated in alum (Figure 6A). We also included an additional group that was immunized with CSP9_NVDP_, a recombinant protein that also had a 9-mer repeat but included three NVDP repeats (Figure S1), as it has been suggested that antibodies that preferentially bind (NANPNVDP)_n_ might confer superior protection to pure (NANP)_n_ binding antibodies (Wang et al., 2020).

Overall, IgG responses to PfCSP (as measured by ELISA on CSP27-coated plates) were similar in magnitude across all immunized groups (Figure 6B). Because differences between immunized mice and control mice are large (as are differences between pre-immune and post-immune sera), but are not of particular interest, this group was removed from subsequent analysis as was the pre-immune time point to avoid skewing the statistical models. Examination of the specificity of the IgG responses at the domain level revealed significant differences in responses to the different immunogens, as follows: CSP9 and CSP9_NVDP_ induced significantly stronger IgG responses to CSP_Nterm_ and CSP_Cterm_ than CSP27 (Figures 6C and 6D). Surprisingly, however, CSP_Repeat_-specific antibody responses were similar in all groups after the third dose (Figure 6E). We also performed more specific ELISAs with peptides corresponding to the regions bound by 5D5, the CSP_Nterm_/CSP_Repeat_ junction, and the minor NANPNVDP repeat (Figure S5A). We found that the truncated CSP9 and CSP9_NVDP_ induced stronger responses to the 5D5 peptide that lies within the CSP_Nterm_ (Figure S5B) but that responses to the junction peptide were similar across all immunogens (Figure S5C).

Finally, we assessed the ability of immunization with CSP27, CSP9, and CSP9_NVDP_ to protect against live parasite challenge. At 5 weeks after the final immunization, mice were challenged by the bites of *Anopheles* mosquitoes infected with Pb-PfSPZ parasites expressing GFP. In three experiments, parasite 18S rRNA was measured in the livers of mice 42 h after challenge by RT-PCR. In an additional two experiments, sterile protection was measured by the assessment of the number of days until mice became patent (defined as >0.1% parasitemia as assessed by flow cytometry).

As assessed by RT-PCR, CSP9_NVDP_ immunization did not significantly reduce parasite burdens, whereas immunization with either CSP27 or CSP9 did (Figure 6F). CSP27-immunized mice had significant reductions in their parasite burdens, with 6/15 mice having no detectable parasite 18S rRNA in the liver. CSP9-immunized mice had even lower median levels of parasite 18S rRNA, and the proportion of mice with no detectable parasite 18S rRNA was even higher (10/15). In the two experiments assessing sterile immunity, immunization with CSP9_NVDP_ again did not confer any detectable protection, but CSP9 and CSP27 immunization both protected the majority of mice (92% and 73%, respectively; Figure 6G). We were intrigued by the fact that in both sets of experiments CSP9 conferred superior protection to CSP27, although in no individual experiment was this difference statistically significant. Accordingly, we examined the proportions of mice in each group that were protected (i.e., having no detectable parasites or *P. berghei* 18S rRNA) across all five experiments (Figure 6H). Although this analysis clearly confirmed that immunization with CSP9 and CSP27 both conferred robust protection, the difference between these two groups showed only a trend toward significance (p = 0.060 by Fisher’s exact test).

The lack of protection by the CSP9_NVDP_ construct was surprising to us, especially as this protein and CSP27 both induced significantly higher absolute levels of antibodies to the minor repeat (Figure S5D) and a higher ratio of minor to major repeat binding antibodies (Figure 6I). However, these ELISAs cannot distinguish antibodies that have true dual binding capacity that have been shown to be the most potent sporozoite-neutralizing antibodies identified to date (Wang et al., 2020).

### Distinct immune repertoires respond to the different domains of CSP

**Figure 7:**
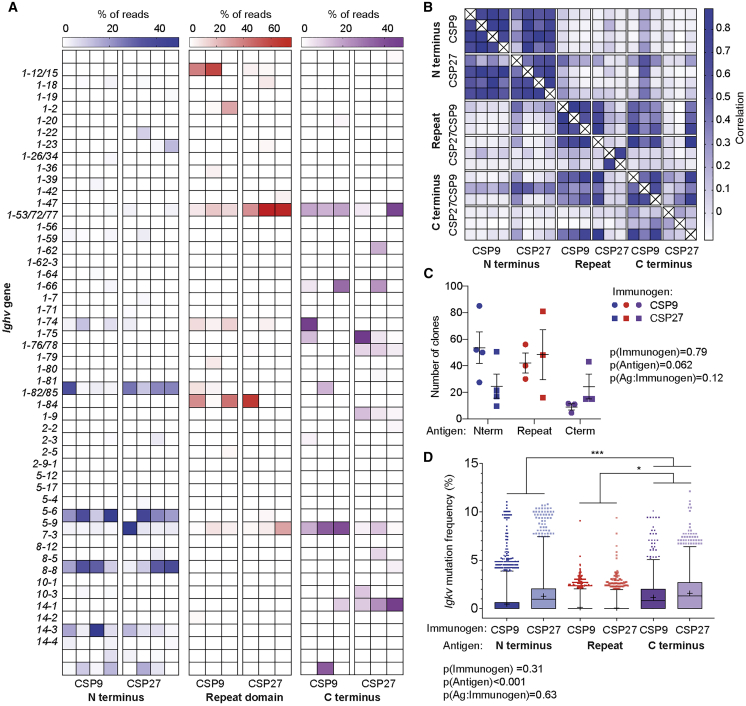
Distinct Ighv gene use by B cells responding to different domains of CSP CSP_Nterm_-, CSP_Repeat_-, and CSP_Cterm_-specific B cells were sorted from mice immunized 10 days previously with CSP27 or CSP9 in alum, and the heavy and light chain Ig genes were amplified with degenerate primers and sequenced. In each of the 2 groups, 10 mice were immunized in total; for each antigen, whole spleens from 3–4 mice were sorted to obtain specific B cells. (A) Ighv gene use among B cells specific for each antigen. (B) Correlation of Ighv gene use for the different antigens calculated. (C) Number of unique clones identified among B cells responding to each antigen; mean ± SEM shown. (D) Mutation frequency among unique reads in the Igkv genes used by B cells responding to each antigen; box shows interquartile range, and whiskers show 1%–99% range. Data in (C) and (B) were analyzed via two-way ANOVA, with mouse included in the model as fixed factors. ANOVA p values are listed below or adjacent to each graph. Pairwise comparisons were performed using a Tukey post-test, and significant values are represented as symbols; ^∗^p < 0.05, ^∗∗^p < 0.01, ^∗∗∗^p < 0.001.

To gain insight into the quality and diversity of the response to each individual domain, we examined the Ig gene use of responding B cells. Accordingly, 10 days after immunization with either CSP27 or CSP9, GC B cells specific for each antigen were enriched by magnetic bead purification and sorted for VDJ sequencing as described previously (Arnaout et al., 2011; Fisher et al., 2017). Examination of the *Ighv* gene use showed that the responses to each domain were distinct (Figure 7A), but V region use to particular domains was similar among the replicate animals (Figure 7B). Similar results were obtained for the *Igkv* gene (Figure S6). The response to CSP_Nterm_ was dominated by *Ighv5-4*, *Ighv8-2*, and *Ighv1-81*. Of note, *Ighv1-81* is the *Ighv* gene used by the 5D5 mAb. *Ighv1-72* and/or the related *Ighv1-53* was prominent in the response to the CSP_Repeat_ by all mice, but the use of this gene was particularly pronounced in mice immunized with CSP27. Mice immunized with CSP9 showed a more prominent use of other genes, notably *Ighv1-18* and *Ighv1-82/85*. The response to the CSP_Cterm_ was more diverse; however, responses were still highly correlated between different animals.

Interestingly, the number of clones responding to each individual antigen was small (<100; Figure 7C), which is consistent with our previous data suggesting that the number of precursors is in excess compared to the number of responding cells (Figures 1 and 2). Although the number of responding clones did not differ significantly for any antigen, there was a trend toward more cells being recruited to the CSP_Nterm_ response when the truncated CSP9 immunogen was used (p = 0.17; Figure 7C). Last, we examined the mutation rate among B cells responding to the different antigens (Figure 7D). It has previously been observed that CSP_Repeat_ specific from sporozoites and RTS,S-vaccinated humans have low levels of somatic hypermutation (SHM) (Aye et al., 2020; McNamara et al., 2020; Murugan et al., 2018). This could be indicative of an inefficient GC response to this antigen, perhaps driven by overly strong signaling from the repeating epitope (Cockburn and Seder, 2018). In agreement with the previous human data, CSP_Repeat_-specific B cells had lower levels of mutation than B cells specific for the other domains (Figure 7D). However, the level of mutation among CSP_Repeat_-specific B cells was similar in CSP9- and CSP27-immunized mice. This finding suggests either that further truncation may be required to allow for more efficient GC reactions to this antigen or the low level of SHM among B cells responding to this antigen is independent of the repeating nature of the antigen.

## Discussion

Here, we show that the primary driver of the immunodominance of the CSP_Repeat_ over other domains within CSP is the avidity of the binding between long repeats and BCRs on the surface of antigen-specific B cells. Reducing the valency of the CSP_Repeat_ allows the development of stronger B cell and antibody responses to other epitopes. To demonstrate the importance of this observation for vaccination, we used truncated CSP molecules to immunize against malaria in a pre-clinical model. We found that mice immunized with truncated CSP developed stronger responses to the CSP_Nterm_ and CSP_Cterm_ domains, both of which have been associated with protective antibody responses. Moreover, mice immunized with truncated CSP molecules were protected against live parasite challenge, and the magnitude of this protection was greater than in mice immunized with nearly full-length CSP. Our results provide direct evidence for the decoy hypothesis and suggest an innovative avenue for malaria vaccination.

Our finding that reducing the length of the CSP_Repeat_ allows responses to other antigens to develop suggests that the long repeat not only drives a large response to this antigen itself but also allows it to suppress other responses. The discovery of this immunodomination effect provides direct support for the decoy hypothesis, although the exact mechanism for this effect is unclear. Our observation that CSP27 drives stronger BCR signaling than CSP9 suggests that this antigen may drive a stronger expansion of the B cell response at the outset. An alternative, non-mutually exclusive hypothesis, is that is that CSP molecules carrying long repeats may be readily taken up by CSP_Repeat_-specific B cells, allowing these B cells to outcompete CSP_Nterm_- and CSP_Cterm_-specific B cells for T cell help (Batista and Neuberger, 2000; Cheng et al., 1999). In agreement with the latter hypothesis, it has been previously shown that competition for restricted T cell help can suppress responses to rare or subdominant epitopes (Woodruff et al., 2018).

Although CSP immunodominance is mainly driven by the ability of the CSP_Repeat_ to bind BCRs multivalently, we cannot completely exclude a role for precursor number in the B cell immunodominance hierarchy. Our data agree with previous studies that found a relationship between B cell precursor number and the subsequent GC response (Abbott et al., 2018; Dosenovic et al., 2018). However, surprisingly, the enhanced response we observed did not occur at the expense of responses to other epitopes. Thus, the presence of a large numbers of precursors specific for the CSP_Repeat_ domain would not necessarily be to the detriment of responses to other epitopes. We hypothesized that the lack of immunodomination after the transfer of large numbers of CSP_Repeat_-specific B cells to wild-type mice may be due to an excess of precursor B cells for the competing NP antigen (Weisel et al., 2016). We therefore created a situation in which the number of NP precursors could be limited further and large excesses of CSP_Repeat_-specific Igh^g2A10^ cells could be transferred. However, even in this situation, there was, at most, limited suppression of the B1-8^hi^ cell response. One hypothesis is that the B1-8^hi^ cells have high initial affinity (5 × 10^−6^ M) for NP (Shih et al., 2002a) and so may easily compete with other cells. However, the estimated affinity of Igh^g2A10^ cells for CSP27 (1.33 × 10^−7^ M) is also high (McNamara et al., 2020), so they should be able to efficiently compete with the B1-8^hi^ cells. Nonetheless, we cannot exclude the possibility that rare low-affinity cells may be suppressed by larger numbers of high-affinity cells responding simultaneously to different epitopes.

Our vaccination approach provides a strategy to rebalance the immunodominance hierarchy within CSP to favor a more diverse and potentially protective response. Similar principles are being applied to vaccines for influenza and HIV. For HIV, there is strong interest in developing multivalent immunogens targeting rare bnAb germline precursors to enhance the frequency of these cells (Jardine et al., 2013; McGuire et al., 2013). For influenza, a possible vaccine strategy is to develop HA immunogens lacking the immunodominant, but variable, head domain (Angeletti et al., 2019). One approach that might be possible, based upon studies in model systems, would be to delete B cells specific for non-protective epitopes by injecting pure epitopes not linked to a T cell epitope (Silva et al., 2017). For CSP-based malaria vaccines, it is probably not desirable to remove the responses to the repeat altogether, as antibodies targeting this domain are clearly protective; however, a rebalancing of the immune response may be desirable.

Although the truncated CSP9 immunogen induced a more diverse immune response than the longer CSP27 molecule, this may not be the sole mechanism of enhanced protection. A previous study using immunization with repeat-only vaccines found the optimal repeat length was just five NANP repeats (Langowski et al., 2020). In this case, other domains of CSP were not present, and thus, the mechanism of protection cannot be the induction of a more diverse immune response. One possibility is that competition for antigens among CSP_Repeat_-specific GC B cells will be more intense if the antigen can crosslink fewer BCRs. In agreement with this, numerous studies have identified few mutations in CSP-specific B cells, suggesting SHM is inefficient (Murugan et al., 2018; Tan et al., 2018). Another association with protection by CSP9 is the lack of NVDP repeats; however, this contradicts findings that NVDP cross-reactive-binding antibodies may be more protective (Oyen et al., 2020; Wang et al., 2020). Notably, though, the current RTS,S vaccine contains a somewhat truncated (18-mer), pure NANP repeat (Casares et al., 2010).

Collectively, our results provide insights into the factors that drive the immunodominance of different B cell epitopes; in particular, we show that repeat epitopes can induce larger responses at the expense of subdominant epitopes. Our results suggest that truncated CSP molecules carrying all domains of the protein may be a promising approach for the development of a next-generation CSP-based vaccine for malaria.

## STAR★Methods

### Key resources table

**Table:** 

REAGENT or RESOURCE	SOURCE	IDENTIFIER
**Antibodies**		

TruStain fcX antibody: Rat anti mouse CD16/32 (Clone: 93)	Biolegend	Catalog # 101320; RRID: AB_1574975
Anti-mouse CD38 (Clone: 90) APC A700	eBioscience	Catalog # 56-0381-82; RRID: AB_657740
Anti-mouse IgM (Clone: II/41) APC Cy7	Invitrogen	Catalog # 47-5790-82; RRID: AB_2573984
Anti-mouse CD19 (Clone: 1D3) BUV395	BD Horizon	Catalog # 563557; RRID: AB_2722495
Anti-mouse IgD (Clone: 11-26c.2a) BV605	Biolegend	Catalog # 405727; RRID: AB_2562887
Anti-mouse B220 (Clone: RA3-6B2) BV605	Biolegend	Catalog # 103244; RRID: AB_2563312
Anti-mouse CD45.1 (Clone: A20) FITC	Biolegend	Catalog # 110706; RRID: AB_313495
Anti-mouse CD45.2 (Clone: 104) PE	Biolegend	Catalog # 110716; RRID:AB_313444
Anti-mouse GL7 (Clone: GL-7) PacificBlue	Invitrogen	Catalog # 48-5902-82; RRID: AB_10870775
Anti-mouse CD138 (Clone: 281-2) PE Cy7	Biolegend	Catalog # 142514; RRID: AB_2562198
Anti-mouse CD3 (Clone: 17A2) PerCPCy5.5	Biolegend	Catalog # 100218; AB_1595492
Anti-mouse CD11b (Clone: M1/70) PerCPCy5.5	Biolegend	Catalog # 101228; RRID: AB_893232
Anti-mouse CD11c (Clone: N418) PerCPCy5.5	Biolegend	Catalog # 117328; RRID: AB_2129641
Anti-mouse Ly-6G/Ly- 6C (GR1) (Clone: RB6-8C5) PerCPCy5.5	Biolegend	Catalog # 108428; RRID: AB_893558
Polyclonal anti-mouse IgG (H+L) HRP	SeraCare	Catalog # 5220-0341; Ref #074-1806
mab:5d5	Leidos	N/A
mab:CIS43	Vaccine Research Centre: Kisalu et al., 2018	N/A
mab:2A10	Genscript	N/A
mab:15	Vaccine Research Centre	N/A
mAb:1710	Hospital for Sick Children Research Institute, Canada: Scally et al., 2018	N/A
mab:3919	Hospital for Sick Children Research Institute, Canada: Scally et al., 2018	N/A
NP (14) BSA	Biosearch	Catalog # N-5050-10
Streptavidin, APC Conjugate (SA-APC)	Invitrogen	Catalog # SA1005
Streptavidin, R-Phycoerythrin Conjugate (SA-PE)	Invitrogen	Catalog # S866
Anti-PE microbeads	Miltenyi Biotech	Catalog # 130-048-801; RRID: AB_244373
Rabbit, polyclonal anti-His-tag antibody (Polyclonal)	GenScript	Catalog # A00174-40; RRID:AB_914703

**Chemicals, peptides, and recombinant proteins**		

7AAD Cell Viability Dye	Biolegend	Catalog # 420404
Platinum taq polymerase	Invitrogen	Catalog # 14966001
dNTP mix	Fermentas	Catalog # R0192
DTT	Invitrogen	Catalog # 18064-014
Magnesium chloride (MgCl2)	Sigma-Aldrich	Catalog # M8266
CloneAmp HiFi PCR Premix	Clontech	Catalog # 639298
Superscript II reverse transcriptase	Invitrogen	Catalog # 18064-014
Power SYBR Green PCR Master Mix	Biosystems	Catalog # 4367659
iScript cDNA Synthesis Kit	BioRad	Catalog # 170-8891
*P. falciparum* Repeat (NANP)4-biotin peptide	Biomatik	Custom order
*P. falciparum* Repeat (NANP)9-biotin peptide	Biomatik	Custom order
*P. falciparum* R1+ biotin peptide	Biomatik	Custom order
*P. falciparum* N81-91 biotin peptide	Biomatik	Custom order
*P. falciparum* 5D5 biotin peptide	Biomatik	Custom order
*P. falciparum* NVDP biotin peptide	Biomatik	Custom order
*P. falciparum* N terminus biotin peptide	Vaccine Research Centre: Kisalu et al., 2018	N/A
*P. falciparum* C terminus biotin peptide	Vaccine Research Centre: Kisalu et al., 2018	N/A
Recombinant *P. falciparum* CSP27	Genescript	Custom order
Recombinant *P. falciparum* CSP9 NVDP	Genescript	Custom order
Recombinant *P. falciparum* CSP9	Genescript	Custom order
NIP-Ɛ-Aminocaproyl-Osu	Biosearch Technologies	Catalog # N-1110-100
RPMI Medium	GIBCO	Catalog #11875-093
Ammonium-Chloride-Potassium (ACK) lysis buffer	GIBCO LifeTech	Catalog # A10492-01
FACS buffer: 2% Fetal Bovine Serum (FBS), 2 mM Ethylenediaminetetraacetic acid (EDTA) in PBS	Sigma, Invitrogen	Catalog # F9423, Catalog # AM9261
Washing buffer: 0.05% Tween 20 in PBS	Sigma	Catalog # P1379
Blocking buffer: 1% Bovine Serum Albumin (BSA) in PBS	Sigma	Catalog # A7906
Detection solution: KPL peroxidase 2 component (1:1)	SeraCare	Catalog # 5120-0032
Stop solution: 1% Sodium Dodecyl Sulfate in PBS	Sigma	Catalog # L4390-250G
Dimethylformamide	Sigma	Catalog # D-4551
Sodium Azide	Sigma	Catalog # S2002

**Critical commercial assays**		

Agencourt Ampure XP beads	Beckman Coulter	Catalog # 63881
Nextera® XT DNA Library Preparation Kit	Illumina	Catalog # FC-131-1096
Nextera® XT 24 Index Kit	Illumina	Catalog # FC131-1001
PicoPure RNA Extraction Kit	Applied Biosystems	Catalog # KIT0204
Anti-PE microbeads	Miltenyi Biotech	Catalog # 130-048-801
LS Columns	Miltenyi Biotech	Catalog # 130-042-401

**Deposited data**		

Heavy and light chain sequences of B cells specific to different CSP epitopes	This paper	PRJNA681645, SUB8631295

**Experimental models: Organisms/strains**		

Mouse: C57BL/6NCrl	Australian Phenomics Facility, Canberra	N/A
Mouse: Ighg2A10	McNamara et al., 2020	N/A
Mouse: MD4 (C57BL/6-Tg(IghelMD4)4Ccg	Goodnow et al., 1989	N/A
Mouse: B1-8	Shih et al., 2002b	N/A
Parasite: Pb-PfSPZ	*P. berghei* sporozoites expressing *P. falciparum* CSP (Espinosa et al., 2017)	N/A

**Oligonucleotides**		

*P. berghei* 18S RNA, Forward: 5′ – AAGCATTAAATAAAGCGAATACATCCTTAC – 3′,	Sigma-Aldrich,	Custom order
*P. berghei* 18S RNA, Reverse: 5′ – GGAGATTGGTTTTGACGTTTATGTG – 3′	Sigma-Aldrich,	Custom order
Glyceraldehyde 3-phosphate dehydrogenase (GapDH), Forward: 5′ – GTTGTCTCCTGCGACTTCA – 3′,	Sigma-Aldrich,	Custom order
Glyceraldehyde 3-phosphate dehydrogenase (GapDH), Reverse: 5′ – GGTGGTCCAGGGTTTCTTA – 3′	Sigma-Aldrich,	Custom order
Heavy chain VDJ Primer, Forward: (moVH-Fr3 for1- IlluAd) 5′-TCGTCGGCAGCGTCAGATGTGTATAA GAGACAGAAGTTCAAGGGCAAGGCC-3′,	Sigma-Aldrich,	Custom order
Heavy chain VDJ Primer, Forward: (moVH-Fr3 for2- IlluAd) 5′-TCGTCGGCAGCGTCAGATGTGTATAAGA GACAGCTCCAAGAGCCAAGTTTTCTT-3′	Sigma-Aldrich,	Custom order
Heavy chain VDJ Primer, Forward: (moVH-Fr3 for3- IlluAd) 5′-TCGTCGGCAGCGTCAGATGTGTATAA GAGACAGCAATCTCCAAGGATACCTCCA-3′	Sigma-Aldrich,	Custom order
Heavy chain VDJ Primer, Forward: (moVH-Fr3 for4- IlluAd) 5′-TCGTCGGCAGCGTCAGATGTGTATAAG AGACAGCGITTCACCATCTCCAGAGA-3′	Sigma-Aldrich,	Custom order
Heavy chain VDJ Primer, Reverse: (moVH-Fr3 rev1- IlluAd) 5′-GTCTCGTGGGCTCGGAGATGTGTATAAG AGACAGCTTACCTGAGGAGACGGTGAC-3′	Sigma-Aldrich,	Custom order
Heavy chain VDJ Primer, Reverse: (moVH-Fr3 rev2- IlluAd) 5′-GTCTCGTGGGCTCGGAGATGTGTATAAGA GACAGAGGACTCACCTGAGGAGAC-3′	Sigma-Aldrich,	Custom order
Light chain VJ Primers, Forward: 5′-TCGTCGGCAGCGTCAGATGTGTATAAG AGACAGGAYATTGTGMTSACMCARWCTMCA-3′	Sigma-Aldrich,	Custom order
Light chain VJ Primers, Reverse: 5′-GTCTCGTGGGCTCGGAGATGTGTATAAG AGACAGACTGAGGCACCTCCAGATGTT-3′	Sigma-Aldrich,	Custom order

**Software and algorithms**		

Adobe Illustrator	Adobe	RRID: SCR_010279
FlowJo	FlowJo	RRID: SCR_008520
GraphPad Prism	GraphPad Software	RRID: SCR_002798
R (version)	The R Foundation for Statistical Computing	RRID: SCR_001905
MiXCR	Bolotin et al., 2015	RRID:SCR_018725
VDJ tools	Shugay et al., 2015	RRID:SCR_005475
immunarch		https://doi.org/10.5281/zenodo.3367200
IMGT tools	(Brochet et al., 2008)	N/A

### Resource availability

#### Lead contact

Further information and requests for resources and reagents should be directed to and will be fulfilled by the lead contact, Ian Cockburn (ian.cockburn@anu.edu.au).

#### Materials availability

No new materials were generated in this study.

#### Data and code availability

Sequencing datasets generated during the study have been deposited at NCBI (https://www.ncbi.nlm.nih.gov/bioproject/?term=PRJNA681645), and has the BioProject ID; PRJNA681645 and SubmissionID; SUB8631295. Otherwise the published article includes all datasets generated/analyzed during this study.

### Experimental model and subject details

#### Ethics statement

All animal procedures were approved by the Animal Experimentation Ethics Committee of the Australian National University (Protocol numbers: A2016/17; 2019/36). All research involving animals was conducted in accordance with the National Health and Medical Research Council’s Australian Code for the Care and Use of Animals for Scientific Purposes and the Australian Capital Territory Animal Welfare Act 1992.

#### Mice

C57BL/6 mice, MD4 (Goodnow et al., 1989), B1-8 mice (Shih et al., 2002b) and Ighg2A10 (McNamara et al., 2020) were bred in-house at the Australian National University. All mice were on a C57BL/6 background. Mice used for the experiment were 5 to 8 weeks, and they were age matched for each experiment groups. Mostly female mice were used throughout the experiments, and were bred and maintained under specific pathogen free conditions in individually ventilated cages at the Australian National University.

#### Parasites

*P. berghei* parasites engineered to express *P. falciparum* CSP in place of the endogenous P. berghei CSP molecule (Pb-PfSPZ) (Espinosa et al., 2017) were used throughout the study. Parasites were maintained by serial passage through *Anopheles stephensi* mosquitoes.

Mice were immunized IV with 5 × 10^4^ irradiated (15kRad) Pb-PfSPZ dissected by hand from the salivary glands of *Anopheles stephensi* mosquitoes.

### Method details

#### Proteins and Immunizations

For immunization with CSP27, CSP9 or CSP9NVDP, or CSP27-NP conjugates 30 μg protein or as described in the relevant figure legend was emulsified in Imject Alum according to the manufacturer’s instructions (ThermoFisher Scientific) and delivered intra-peritoneally.

#### Lymphocyte isolation and flow cytometry

Spleens were collected from mice after cervical dislocation, and single cell suspension was prepared after passing through a 70 μM cell strainer into FACS buffer. Cells were briefly blocked for 30 minutes using 1μg/mL Streptavidin and 10 μg/mL TruStain fcX antibody diluted in FACS buffer. An antibody master mix was prepared in FACS buffer to stain the surface antigens of cells as per standard protocol using antibodies and B cell tetramers (prepared in house) for 30 minutes in dark. Cells were then lyzed using ACK lysis buffer and washed twice before staining with 1% 7AAD as Live/dead dye. Samples were acquired with a BD Fortessa or X20 flow cytometer, and data was analyzed using FlowJo software. The universal gating strategy is shown Figure S2, after which the further gating to study the B cells of interest is provided in the results.

For adoptive transfer experiments with Ighg2A10 and B1-8 cells, single cell suspensions of splenocytes were prepared and an aliquot used for flow cytometry using probes as described below to determine the proportion of cells that were antigen-specific B cells. The concentration of splenocytes was then normalized to allow for the transfer of the desired number of antigen specific cells.

#### ELISA measurement of antigen-specific IgG

Blood was collected from mice via either tail vein or retro-orbital bleeds and left to clot overnight at 4 **°**C, and the following day sera was collected by spinning the blood at 2000 *g* for 15 minutes at 4 **°**C. Next, Maxisorp Nunc-Nucleon 96 flat bottom plates were coated with 1 μg/ml streptavidin overnight at 4 **°**C in dark. The following day, plates were washed in wash buffer consisting 0.05% tween20 in PBS, and were incubated for 1 hour with different biotinylated peptides listed in pertaining to diverse sections of CSP protein. The peptide sequences were as follows, CSP_Nterm_: Biotin-Ahx-QEYQCYGSSSNTRVLNELNYDNAGTNLYNELEMNYYGKQENWYSLKKNSRSLGENDDGNNEDNEKLRKPKHKKLKQPADG; CSP_Repeat_: Biotin-Ahx-NANPNANPNANPNANPNANPNANP NANPNANPNANP, and CSP_Cterm_: Biotin-Ahx-NKNNQGNGQGHNMPNDPNRNVDENANANSAVKNNNNEEPSDKHIKEYLNKIQNSSTEWSPCSVTCGNGIQVRI KPGSANKPKDELDYANDIEKKICKMEKCS. Additional peptides also carrying a Biotin-Ahx linker are described in Figure S6. Wells were blocked for 1 hour before incubating with initial sera (dilution of 1/100 times, followed by 1 in 4 serial dilutions down the plate) for one hour. After washing, anti IgG detection antibody conjugated to HRP was diluted 1/2000 times and was incubated for one hour. The plates were washed, then developed with Peroxidase Substrate Kit for 15 minutes, and the reaction was stopped using 50 μL/ well stop solution, consisting of 10% SDS in PBS. Absorbance at 405 nm (A405) was measured using an Infinite PRO Tecan plate reader, and the data was expressed as area under the curve (AUC) calculated in Prism 7 from the log(dilution) on the x axis and the A405 on the y axis, fitting a sigmoidal curve.

#### Quantitation of antigen specific naive precursors

Splenocytes was harvested from naive C57BL/6 or MD4 mice. Cells were then incubated for 30 minutes with a mixture of tetramers consisting full length recombinant CSP conjugated to PE and domain specific region of CSP, conjugated to APC. The cells were washed to remove unbound tetramers, and were enriched for PE after incubating with anti-PE microbeads and passing over the LS magnetic column (Miltenyi) according to the manufactures recommendation. Subsequently, antibody staining was performed on the enriched cells as per established protocol. Prior to acquisition on a BD X-20 flow cytometer, 1 × 10^4^ CountBright absolute counting beads (Invitrogen) were added, to normalize the number of tetramer specific cell events obtained in each sample.

#### Conjugation of NP to CSP

CSP27 or CSP9 was initially concentrated using an Amicon ultra-15 centrifugal filter unit (10 kD molecular weight cut-off, MWCO). Briefly, the filter was washed by spinning 10 mL of MQ at 4000 *g* for 20 minutes at 4 **°**C then discarding the flow through and any retained water. 2 mg of CSP27 or 1 mg CSP9 was added to the membrane and made up to 10 mL in 3% NaHCO_3_, then spun at 4000 *g* for 60 minutes at 4 **°**C. The flow-through was discarded and the retained protein made up to 10 mL in 3% NaHCO_3_, then respun at 4000 *g* for 60 minutes at 4 **°**C. The flow-through was discarded and the retained protein was made up to final volume of 1 mL per mg of original starting material in 3% NaHCO^+^. This was then transferred into a pre-soaked 3.5 kD MWCO dialysis tubing and dialysed overnight, then for 4 hours in 1 L 3% NaHCO_3_ at 4 **°**C.

Next, NP-ε-Aminocaproyl-Osu (NP-CAP-Osu) was dissolved in DMF to a final concentration of 10 mg/mL. We determined empirically that a 2:1 ratio of NP:CSP in the final conjugated product required a 20:1 ratio of NP:CSP at the conjugation step. For other NP:CSP ratios (6:1 and 10:1) this 10-fold increase during conjugation was also used; i.e., 60:1 and 100:1, respectively. The dialysed CSP and required NP-CAP-Osu were combined in sterile 2 mL tubes, covered with foil to protect from light, then rotated for 4 hours at RT to conjugate. The conjugated products were then dialysed in 1 L 3% NaHCO_3_ at 4 **°**C for 5 hours, overnight then 4 hours before dialysis in 1 L PBS for 4 hours and overnight. Conjugated products were stored at 4 **°**C with 0.1% sodium azide to prevent bacterial growth.

#### Determination of NP:CSP ratio

Since NP-CAP-Osu has an absorption maximum of 430nm with extinction coefficient of 4230, the concentration of NP was determined via NanoDrop. Briefly, 1.5 μL aliquots of CSP27-NP/ CSP9-NP were analyzed on a NanoDrop ND-1000 Spectrophotometer to measure protein at 280 nm and NP dye at 430 nm. An average of two reads was used to calculate the concentration of NP.

The absorbance of NP influenced the absorbance of proteins at 280 nm, therefore NanoDrop was not an accurate method for quantification of protein concentration (CSP27/CSP9) in the conjugated products. Instead, the concentration of CSP27 or CSP9 was determined by sandwich ELISA, taking advantage of histidine-tags (His-tag) attached to both CSP27 and CSP9 (Figure S1). Briefly, 96-well Maxisorp Nunc-Nucleon plates were coated overnight with 1 μg/mL of anti His-tag antibody. The following day, plates were washed and blocked with 1% BSA for 1 hour. The NP-CSP conjugated products were initially diluted 1/100 in 1% BSA along with a 1/100 dilution of a CSP27 or CSP9 standard with known concentration, and serially diluted 1 in 5 times down the plate. Next, a 1/2000 dilution of 2A10 primary antibody (Zavala et al., 1983) was incubated for one hour, washed and incubated with secondary antibody (Anti IgG detection antibody conjugated to HRP) for one hour. After washing, the plates were developed with Peroxidase Substrate Kit for 15 minutes and read at 405 nm using a Tecan Infinite 200Pro plate reader. The reaction was stopped using 50 μL/ well stop solution, consisting of 10% SDS in PBS. The concentration of CSP27 or CSP9 in the conjugated products was interpolated from a sigmoidal standard curve of CSP27 or CSP9.

#### Preparation of B cell tetramers

To detect B cells to specific epitopes in CSP we used tetramers based on peptide probes described above for the CSP_Repeat_, CSP_Nterm_, or CSP_Cterm_ region conjugated to phycoerythrin (PE) or allophycocyanin (APC). The tetramers were generated by mixing biotin-conjugated peptides with streptavidin-conjugated PE or streptavidin-conjugated APC in a 4:1 molar ratio. Briefly, 2.17 nM of peptide was made up to 50 μL in PBS. Then 8.68 nM of PE or APC were added in 4 equal aliquots every 15 minutes, incubating at room temperature (RT) in darkness between aliquots. Tetramers were stored at 4°C in dark until use.

NP-specific B cells were detected using 4-hydroxy-5-iodo-3-nitrophenol (NIP) conjugated to PE or APC. Briefly, 1 mg of Native R-Phycoerythrin protein or Natural Allophycocyanin protein were transferred into pre-soaked 3.5 kD MWCO dialysis tubing and dialysed for 5 hours, overnight, then for 4 hours in 1 L 3% NaHCO_3_ at 4°C. NIP- ε -Aminocaproyl-Osu (NIP-CAP-Osu) was dissolved in DMF to a concentration of 10mg/mL. The NIP-CAP-Osu was added to the dialysed PE or APC at a ratio of 20 μg:1 mg and rotated at RT for 4 hours protected from light with aluminum foil. The conjugated NP-PE and NP-APC were then dialysed in 1 L 3% NaHCO_3_ at 4°C for 5 hours, overnight then 4 hours before dialysis in 1 L PBS for 4 hours and overnight. NP probes were stored at 4°C in dark till use.

#### Immunization with NP-CSP conjugates

Immunisations were conducted with the following amounts of antigen: NP-CSP27 = 15 μg/mouse, NP-CSP9 = 11.93 μg/mouse. Negative control groups were immunized with vehicle (PBS in alum). Antigens were emulsified in alum (2:1 volumetric ratio antigen: alum) to a total volume of 150 μL per mouse. The resultant solution was vortexed slowly at RT for 30 minutes to ensure the immunisations were fully emulsified. Immunisations were delivered intraperitoneally (IP), 150 μL total delivered in 75 μL aliquots on each side of abdomen into C57BL/6 recipient mice.

#### Immunization with CSP constructs

Three different constructs of CSP were used for immunization, they include CSP9NVDP, CSP9, and CSP27 (Figure S1). C57BL/6 recipient mice were randomly separated in four groups, three of them had 15 mice per group, and were immunized with one of the CSP constructs. The fourth group had 11 mice and was the negative control. Each mouse received 30 μg of a CSP construct. These were emulsified in alum to a 2:1 volumetric ratio of antigen: alum and a resultant solution of 200 μL, before vortexing at RT for 30 minutes to ensure complete emulsification. Mouse were then IP immunized with 200 μL, 100 μL on left and right side, respectively. The immunisation regimen consisted of one priming and two boaster doses, each separated by an interval of 5 weeks. One day before immunization, blood was collected from mice via tail vein or retro-orbital bleeds for assessing antibody response. 2 weeks after the mice received their final booster, they were challenged via mosquito bite.

#### Challenge of mice via Anopheles stephensi bites

Controlled malaria infection challenge was performed via bite of *Anopheles stephensi* mosquitoes infected with Pb-PfSPZ a *P. berghei* parasite strain that expresses *P. falciparum* CSP (Espinosa et al., 2017). Also, the parasites express GFP or mCherry, allowing infected mosquitoes to be visually identified via microsopy, and 5 positive mosquitoes were sorted onto separate containers. These mosquitoes were fed with sucrose for the first 6 hours and then with water solution a day before the challenge. The following day, mice were anaesthetised and placed on top of the containers to allow the mosquitoes to blood feed for 30 minutes. In some experiments, 42 hours post mosquito bite, mice were euthanised via cervical dislocation and liver was collected, washed twice in PBS, and homogenized in 4 mL denaturing working stock. In other experiments mice were monitored daily for the presence of parasites in the blood which was detected via flow cytometry of blood collected via tail nick. In this case a mouse was considered patent in the day the proportion of RBCs that were GFP^+^ exceeded 0.1%.

#### Quantification of liver parasite burden

To extract parasite rRNA, 60 μL of 2 M Sodium Acetate was added to a 600 μL aliquot of homogenized liver and vortexed to mix. Then 750 μL Acid Phenol:Chloroform was added and vortexed before incubating on ice for 15 minutes. The samples were spun at 15000 *g* for 20 minutes at 4°C and the upper aqueous phase transferred into a clean 1.5 mL tube. The RNA was precipitated via addition of 400 μL isopropanol, vortexing then incubating at −20°C for 1 hour. The RNA was pelleted at 15000 *g* for 20 minutes at 4°C then the pellet washed twice with 1 mL cold 70% ethanol (EtOH) then dried for 10 minutes before resuspension in ultra-pure water. RNA concentration was measured via Nanodrop, and was diluted to make 100 μL aliquots at 50 ng/μL and stored at 4°C.

cDNA was synthesized from the RNA using iScript cDNA Synthesis Kit according to the manufacturer’s protocol. Briefly, each sample was run in a 20 μL reaction in individual dome-capped PCR tubes containing 10 μL ultra-pure water, 4 μL 5 x iScript Reaction Mix, 1 μL iScript Reverse Transcriptase and 5 μL RNA (50 ng/μL). The samples were run on an Eppendorf ProS Mastercycler at 25°C for 5 minutes, 42°C for 30 minutes, 85°C for 5 minutes then hold at 4°C.

RT qPCR was run using Power SYBR Green PCR Master Mix. Briefly, a PCR mastermix was made for all samples plus 2 no template controls (NTC) and 5 standards (STD) with the following volume per one reaction: 4.4 μL ultra-pure water, 5 μL Power SYBR Green PCR Master Mix, 0.05 μL *P. berghei* forward primer and 0.05 μL *P. berghei* reverse primer. 38 μL aliquots of mastermix were transferred into 1.5 mL tubes for each condition; 2 NTC, 5 STD and x cDNA samples. For DNA templates for *P. berghei* 18S qPCR 2 μL of the following was added for each condition; NTC – ultra-pure water, STD – plasmid standards (10^7^, 10^6^, 10^5^, 10^4^ and 10^3^), samples – cDNA. The PCR was plated in triplicates of 10 μL/well in a MicroAmp 384 well reaction plate. The plate was sealed with MicroAmp optical adhesive film and spun at 500 *g* for 15 s to ensure all samples were at the bottom of each well. The qPCR was run on a 7900HT Fast Real-Time PCR System using the following conditions; 50°C for 2 minutes, 95°C for 10 minutes then 40 cycles of 95°C for 15 s and 60°C for 1 minute followed by 95°C for 15 s and 60°C for 15 s. The above qPCR reaction was repeated for glyceraldehyde 3-phosphate dehydrogenase (GapDH) using GapDH primers. However, the standards consisted instead of a pool of 2 μL cDNA from each sample that was serially diluted for the following concentrations; 1.0, 0.5, 0.25, 0.125, 0.0625. qPCR data was read using sDS2.4 software, *P. berghei* 18S was normalized to the GapDH reference gene before further calculating means in Prism 7.

#### Calcium flux measurement

CSP_Repeat_-specific B cells were FACS purified from Igh^g2A10^ knock-in mouse splenocytes (McNamara et al., unpublished data), and were cultured in complete RPMI for 16 hours. Then the cells were labeled in RPMI media containing 2 μM/ml Indo-1 and 7AAD for 20 min at 37°C. Following2 washes, the signal at BUV395 channel (Indo-1 bound) and BUV496 channel (Indo-1 free) was collected for 60 s to define baseline Ca^2+^ levels as the ratio of Indo-1 (bound/free). B cells were then stimulated for additional 360 s with either 0.5 μM/ml CSP or 10 μg/ml OVA-HEL. 1 μg/ml ionomycin was used as positive control. Data were collected in a FACS Fortessa instrument (BD) and analyzed using the Kinetics tool in FlowJo software (Tree Star). To analyzed the result, the baseline Ca^2+^ level was defined by the mean value of Indo-1 (bound/free) within 0-60 s. The calcium influx was then calculated by the mean values of every 30 s divided by the baseline value, and the plot was made by the calcium influx versus the mean value of this time frame.

#### Surface Plasmon Resonance

Surface plasmon resonance saturation experiments were performed on a Biacore 8K instrument (GE Healthcare) at 25°C using a Series S Sensor Chip NTA (GE Healthcare) and SPR running buffer (10 mM HEPES, 150 mM NaCl, 50 μM EDTA, 0.05% v/v Tween 20, pH 7.4). Solutions of His_6_-tagged CSP27 and CSP9 were prepared in SPR running buffer at concentrations of 0.1 μg/ml and 0.4 μg/ml, respectively. His_6_-tagged CSP27 and CSP9 were immobilized on separate channels on the sensor chip surface as per the manufacturer’s recommendations: a pre-conditioned chip was first activated with 500 μM NiCl_2_, and a solution of the His_6_-tagged ligand was subsequently passed over the chip using a flow rate of 5 μl/min for 120 s. This yielded approximately 150 RU and 50 RU of immobilized CSP27 and CSP9, respectively. A saturating solution of mAb 2A10 (2 μM in SPR running buffer) was then passed over the chip for 400 s using a flow rate of 30 μl/min, followed by a 400 s dissociation period. The increase in response units (RU) corresponding to ligand immobilization (RU_lig_) and analyte binding (RU_analyte_) in the reference-subtracted (reference = blank surface) sensorgrams was measured, and the binding stoichiometry (*n*, molar ratio of antibody to antigen in the complex under saturating concentrations of mAb 2A10) was estimated using Equation (1) as previously described (Oda and Azuma, 2000), using molecular weights (MW) calculated using ProtParam (Wilkins et al., 1999):CSP27 (35.4 kDa), CSP9 (28.2 kDa) and mAb 2A10 (145.9 kDa).

(1) $$n=\frac{RU_{\mathrm{analyte}}}{RU_{\mathrm{ligand}}}\times \frac{MW_{\mathrm{ligand}}}{MW_{\mathrm{analyte}}}$$

All buffers were filtered and degassed prior to use. Following each cycle, the chip was completely regenerated using sequential washes of 500 mM imidazole, 350 mM EDTA (pH 8.5) and 100 mM NaOH. Each experiment was performed in duplicate (n = 2), on separate channels on the SPR chip.

#### Sequencing of CSP-specific B cells

Mice were intraperitoneally immunized with 30 μg of a CSP construct in alum, and on Day10 the spleens were harvested to prepare single cell splenocytes suspension. Individual mouse splenocytes were then stained with either of the CSP epitope specific tetramer, and were enriched using magnetic LS column (Miltenyi), according to the manufacturer’s directions. Subsequently they were stained with antibody master mix as describer previously, and the Germinal Center B cells were sorted using BD FACs Aria I or II (Becton Dickinson) machine. The RNA was then extracted using the PicoPure RNA Extraction Kit (Applied Biosystems), and on the same day cDNA was prepared using the iScript cDNA synthesis kit (BioRad), and were stored in −20°C until further use. BCR sequenced was amplified, and indexed (Nextera indexing kit, Illumina) as previously described approach (Fisher et al., 2017). Sequencing was performed on the Illumina MiSeq sequencing platform. The obtained paired end sequence reads were aligned to *Mus musculus* reference genome at IMGT (Lefranc, 2001) through MiXCR (Bolotin et al., 2015) to obtain the assembled sequences. Top 99% of these sequences were then filtered to remove non coding, or frameshift mutations in the receptor sequences using VDJ tools (Shugay et al., 2015). In the heavy and the light chain aligned and filtered sequences, we then measured the V gene uses and their repertoire overlap using the immunarch package in R (https://doi.org/10.5281/zenodo.3367200). The mutation frequencies in the light chain were plotted by listing the number of substitution, insertion and deletion observed in the top 99% of the sequences while aligning the raw sequencing reads to the germline variant in IMGT through MiXCR (Bolotin et al., 2015; Lefranc, 2001).

### Quantification and statistical analysis

Statistical analysis was performed in GraphPad Prism for simple analyses without blocking factors; all other analyses was performed in R (The R Foundation for Statistical Computing) with details of statistical tests in the relevant figure legends. All experiments performed had biological and technical replicates, with the numbers given in the legends. No randomization or blinding was performed. No data or outliers were excluded in the study. During experiments where data was pooled, each experiment was included as a blocking factor during the analysis. Significant P value abbreviations were as follows; p < 0.05 = ^∗^, p < 0.01 = ^∗∗^, p < 0.001 = ^∗∗∗^, p < 0.0001 = ^∗∗∗∗^.

## Supplemental information

Document S1. Figures S1–S6

Document S2. Article plus supplemental information

## References

[bib1] Abbott R.K., Lee J.H., Menis S., Skog P., Rossi M., Ota T., Kulp D.W., Bhullar D., Kalyuzhniy O., Havenar-Daughton C. (2018). Precursor Frequency and Affinity Determine B Cell Competitive Fitness in Germinal Centers, Tested with Germline-Targeting HIV Vaccine Immunogens. Immunity.

[bib2] Agnandji S.T., Lell B., Fernandes J.F., Abossolo B.P., Methogo B.G., Kabwende A.L., Adegnika A.A., Mordmüller B., Issifou S., Kremsner P.G., RTS,S Clinical Trials Partnership (2012). A phase 3 trial of RTS,S/AS01 malaria vaccine in African infants. N. Engl. J. Med..

[bib3] Angeletti D., Kosik I., Santos J.J.S., Yewdell W.T., Boudreau C.M., Mallajosyula V.V.A., Mankowski M.C., Chambers M., Prabhakaran M., Hickman H.D. (2019). Outflanking immunodominance to target subdominant broadly neutralizing epitopes. Proc. Natl. Acad. Sci. USA.

[bib4] Arnaout R., Lee W., Cahill P., Honan T., Sparrow T., Weiand M., Nusbaum C., Rajewsky K., Koralov S.B. (2011). High-resolution description of antibody heavy-chain repertoires in humans. PLoS One.

[bib5] Aye R., Sutton H.J., Nduati E.W., Kai O., Mwacharo J., Musyoki J., Otieno E., Wambua J., Bejon P., Cockburn I.A., Ndungu F.M. (2020). Malaria exposure drives both cognate and bystander human B cells to adopt an atypical phenotype. Eur. J. Immunol..

[bib6] Batista F.D., Neuberger M.S. (2000). B cells extract and present immobilized antigen: implications for affinity discrimination. EMBO J..

[bib7] Bolotin D.A., Poslavsky S., Mitrophanov I., Shugay M., Mamedov I.Z., Putintseva E.V., Chudakov D.M. (2015). MiXCR: software for comprehensive adaptive immunity profiling. Nat. Methods.

[bib8] Bongfen S.E., Ntsama P.M., Offner S., Smith T., Felger I., Tanner M., Alonso P., Nebie I., Romero J.F., Silvie O. (2009). The N-terminal domain of Plasmodium falciparum circumsporozoite protein represents a target of protective immunity. Vaccine.

[bib59] Brochet X., Lefranc M., Guidicelli V. (2008). IMGT/V-QUEST: the highly customized and integrated system for IG and TR standardized V-J and V-D-J sequence analysis. Nucleic Acids Res.

[bib9] Casares S., Brumeanu T.D., Richie T.L. (2010). The RTS,S malaria vaccine. Vaccine.

[bib10] Cerami C., Frevert U., Sinnis P., Takacs B., Clavijo P., Santos M.J., Nussenzweig V. (1992). The basolateral domain of the hepatocyte plasma membrane bears receptors for the circumsporozoite protein of Plasmodium falciparum sporozoites. Cell.

[bib11] Cheng P.C., Steele C.R., Gu L., Song W., Pierce S.K. (1999). MHC class II antigen processing in B cells: accelerated intracellular targeting of antigens. J. Immunol..

[bib12] Cockburn I.A., Seder R.A. (2018). Malaria prevention: from immunological concepts to effective vaccines and protective antibodies. Nat. Immunol..

[bib13] Dobaño C., Sanz H., Sorgho H., Dosoo D., Mpina M., Ubillos I., Aguilar R., Ford T., Díez-Padrisa N., Williams N.A. (2019). Concentration and avidity of antibodies to different circumsporozoite epitopes correlate with RTS,S/AS01E malaria vaccine efficacy. Nat. Commun..

[bib14] Dosenovic P., Kara E.E., Pettersson A.K., McGuire A.T., Gray M., Hartweger H., Thientosapol E.S., Stamatatos L., Nussenzweig M.C. (2018). Anti-HIV-1 B cell responses are dependent on B cell precursor frequency and antigen-binding affinity. Proc. Natl. Acad. Sci. USA.

[bib15] Espinosa D.A., Gutierrez G.M., Rojas-López M., Noe A.R., Shi L., Tse S.W., Sinnis P., Zavala F. (2015). Proteolytic Cleavage of the Plasmodium falciparum Circumsporozoite Protein Is a Target of Protective Antibodies. J. Infect. Dis..

[bib16] Espinosa D.A., Christensen D., Muñoz C., Singh S., Locke E., Andersen P., Zavala F. (2017). Robust antibody and CD8^+^ T-cell responses induced by *P. falciparum* CSP adsorbed to cationic liposomal adjuvant CAF09 confer sterilizing immunity against experimental rodent malaria infection. NPJ Vaccines.

[bib17] Ferreira A., Morimoto T., Altszuler R., Nussenzweig V. (1987). Use of a DNA probe to measure the neutralization of Plasmodium berghei sporozoites by a monoclonal antibody. J. Immunol..

[bib18] Fisher C.R., Sutton H.J., Kaczmarski J.A., McNamara H.A., Clifton B., Mitchell J., Cai Y., Dups J.N., D’Arcy N.J., Singh M. (2017). T-dependent B cell responses to Plasmodium induce antibodies that form a high-avidity multivalent complex with the circumsporozoite protein. PLoS Pathog..

[bib19] Goodnow C.C., Crosbie J., Jorgensen H., Brink R.A., Basten A. (1989). Induction of self-tolerance in mature peripheral B lymphocytes. Nature.

[bib20] Imkeller K., Scally S.W., Bosch A., Martí G.P., Costa G., Triller G., Murugan R., Renna V., Jumaa H., Kremsner P.G. (2018). Antihomotypic affinity maturation improves human B cell responses against a repetitive epitope. Science.

[bib21] Ishizuka A.S., Lyke K.E., DeZure A., Berry A.A., Richie T.L., Mendoza F.H., Enama M.E., Gordon I.J., Chang L.J., Sarwar U.N. (2016). Protection against malaria at 1 year and immune correlates following PfSPZ vaccination. Nat. Med..

[bib22] Jardine J., Julien J.P., Menis S., Ota T., Kalyuzhniy O., McGuire A., Sok D., Huang P.S., MacPherson S., Jones M. (2013). Rational HIV immunogen design to target specific germline B cell receptors. Science.

[bib23] Kato Y., Abbott R.K., Freeman B.L., Haupt S., Groschel B., Silva M., Menis S., Irvine D.J., Schief W.R., Crotty S. (2020). Multifaceted Effects of Antigen Valency on B Cell Response Composition and Differentiation In Vivo. Immunity.

[bib24] Kisalu N.K., Idris A.H., Weidle C., Flores-Garcia Y., Flynn B.J., Sack B.K., Murphy S., Schön A., Freire E., Francica J.R. (2018). A human monoclonal antibody prevents malaria infection by targeting a new site of vulnerability on the parasite. Nat. Med..

[bib25] Langowski M.D., Khan F.A., Bitzer A.A., Genito C.J., Schrader A.J., Martin M.L., Soto K., Zou X., Hadiwidjojo S., Beck Z. (2020). Optimization of a *Plasmodium falciparum* circumsporozoite protein repeat vaccine using the tobacco mosaic virus platform. Proc. Natl. Acad. Sci. USA.

[bib26] Lefranc M.P. (2001). IMGT, the international ImMunoGeneTics database. Nucleic Acids Res..

[bib27] McGuire A.T., Hoot S., Dreyer A.M., Lippy A., Stuart A., Cohen K.W., Jardine J., Menis S., Scheid J.F., West A.P. (2013). Engineering HIV envelope protein to activate germline B cell receptors of broadly neutralizing anti-CD4 binding site antibodies. J. Exp. Med..

[bib28] McNamara H.A., Idris A.H., Sutton H.J., Vistein R., Flynn B.J., Cai Y., Wiehe K., Lyke K.E., Chatterjee D., Kc N. (2020). Antibody Feedback Limits the Expansion of B Cell Responses to Malaria Vaccination but Drives Diversification of the Humoral Response. Cell Host Microbe.

[bib29] Murugan R., Buchauer L., Triller G., Kreschel C., Costa G., Pidelaserra Martí G., Imkeller K., Busse C.E., Chakravarty S., Sim B.K.L. (2018). Clonal selection drives protective memory B cell responses in controlled human malaria infection. Sci. Immunol..

[bib30] Nussenzweig R.S., Vanderberg J., Most H., Orton C. (1967). Protective immunity produced by the injection of x-irradiated sporozoites of plasmodium berghei. Nature.

[bib31] Oda M., Azuma T. (2000). Reevaluation of stoichiometry and affinity/avidity in interactions between anti-hapten antibodies and mono- or multi-valent antigens. Mol. Immunol..

[bib32] Olotu A., Fegan G., Wambua J., Nyangweso G., Leach A., Lievens M., Kaslow D.C., Njuguna P., Marsh K., Bejon P. (2016). Seven-Year Efficacy of RTS,S/AS01 Malaria Vaccine among Young African Children. N. Engl. J. Med..

[bib33] Oyen D., Torres J.L., Wille-Reece U., Ockenhouse C.F., Emerling D., Glanville J., Volkmuth W., Flores-Garcia Y., Zavala F., Ward A.B. (2017). Structural basis for antibody recognition of the NANP repeats in *Plasmodium falciparum* circumsporozoite protein. Proc. Natl. Acad. Sci. USA.

[bib34] Oyen D., Torres J.L., Cottrell C.A., Richter King C., Wilson I.A., Ward A.B. (2018). Cryo-EM structure of *P. falciparum* circumsporozoite protein with a vaccine-elicited antibody is stabilized by somatically mutated inter-Fab contacts. Sci. Adv..

[bib35] Oyen D., Torres J.L., Aoto P.C., Flores-Garcia Y., Binter Š., Pholcharee T., Carroll S., Reponen S., Wash R., Liang Q. (2020). Structure and mechanism of monoclonal antibody binding to the junctional epitope of Plasmodium falciparum circumsporozoite protein. PLoS Pathog..

[bib36] Potocnjak P., Yoshida N., Nussenzweig R.S., Nussenzweig V. (1980). Monovalent fragments (Fab) of monoclonal antibodies to a sporozoite surface antigen (Pb44) protect mice against malarial infection. J. Exp. Med..

[bib37] RTS,S Clinical Trials Partnership (2015). Efficacy and safety of RTS,S/AS01 malaria vaccine with or without a booster dose in infants and children in Africa: final results of a phase 3, individually randomised, controlled trial. Lancet.

[bib38] Scally S.W., Murugan R., Bosch A., Triller G., Costa G., Mordmüller B., Kremsner P.G., Sim B.K.L., Hoffman S.L., Levashina E.A. (2018). Rare PfCSP C-terminal antibodies induced by live sporozoite vaccination are ineffective against malaria infection. J. Exp. Med..

[bib39] Schofield L. (1990). The circumsporozoite protein of Plasmodium: a mechanism of immune evasion by the malaria parasite?. Bull. World Health Organ..

[bib40] Schofield L., Uadia P. (1990). Lack of Ir gene control in the immune response to malaria. I. A thymus-independent antibody response to the repetitive surface protein of sporozoites. J. Immunol..

[bib41] Seder R.A., Chang L.J., Enama M.E., Zephir K.L., Sarwar U.N., Gordon I.J., Holman L.A., James E.R., Billingsley P.F., Gunasekera A., VRC 312 Study Team (2013). Protection against malaria by intravenous immunization with a nonreplicating sporozoite vaccine. Science.

[bib42] Shih T.A., Meffre E., Roederer M., Nussenzweig M.C. (2002). Role of BCR affinity in T cell dependent antibody responses in vivo. Nat. Immunol..

[bib43] Shih T.A., Roederer M., Nussenzweig M.C. (2002). Role of antigen receptor affinity in T cell-independent antibody responses in vivo. Nat. Immunol..

[bib44] Shugay M., Bagaev D.V., Turchaninova M.A., Bolotin D.A., Britanova O.V., Putintseva E.V., Pogorelyy M.V., Nazarov V.I., Zvyagin I.V., Kirgizova V.I. (2015). VDJtools: Unifying Post-analysis of T Cell Receptor Repertoires. PLoS Comput. Biol..

[bib45] Silva M., Nguyen T.H., Philbrook P., Chu M., Sears O., Hatfield S., Abbott R.K., Kelsoe G., Sitkovsky M.V. (2017). Targeted Elimination of Immunodominant B Cells Drives the Germinal Center Reaction toward Subdominant Epitopes. Cell Rep..

[bib46] Tan J., Sack B.K., Oyen D., Zenklusen I., Piccoli L., Barbieri S., Foglierini M., Fregni C.S., Marcandalli J., Jongo S. (2018). A public antibody lineage that potently inhibits malaria infection through dual binding to the circumsporozoite protein. Nat. Med..

[bib47] Taylor J.J., Pape K.A., Steach H.R., Jenkins M.K. (2015). Humoral immunity. Apoptosis and antigen affinity limit effector cell differentiation of a single naïve B cell. Science.

[bib48] Triller G., Scally S.W., Costa G., Pissarev M., Kreschel C., Bosch A., Marois E., Sack B.K., Murugan R., Salman A.M. (2017). Natural Parasite Exposure Induces Protective Human Anti-Malarial Antibodies. Immunity.

[bib49] Ubillos I., Ayestaran A., Nhabomba A.J., Dosoo D., Vidal M., Jiménez A., Jairoce C., Sanz H., Aguilar R., Williams N.A. (2018). Baseline exposure, antibody subclass, and hepatitis B response differentially affect malaria protective immunity following RTS,S/AS01E vaccination in African children. BMC Med..

[bib51] Wang L.T., Pereira L.S., Flores-Garcia Y., O’Connor J., Flynn B.J., Schön A., Hurlburt N.K., Dillon M., Yang A.S.P., Fabra-García A. (2020). A Potent Anti-Malarial Human Monoclonal Antibody Targets Circumsporozoite Protein Minor Repeats and Neutralizes Sporozoites in the Liver. Immunity.

[bib52] Weisel F.J., Zuccarino-Catania G.V., Chikina M., Shlomchik M.J. (2016). A Temporal Switch in the Germinal Center Determines Differential Output of Memory B and Plasma Cells. Immunity.

[bib53] White M.T., Verity R., Griffin J.T., Asante K.P., Owusu-Agyei S., Greenwood B., Drakeley C., Gesase S., Lusingu J., Ansong D. (2015). Immunogenicity of the RTS,S/AS01 malaria vaccine and implications for duration of vaccine efficacy: secondary analysis of data from a phase 3 randomised controlled trial. Lancet Infect. Dis..

[bib54] Wilkins M.R., Gasteiger E., Bairoch A., Sanchez J.C., Williams K.L., Appel R.D., Hochstrasser D.F. (1999). Protein identification and analysis tools in the ExPASy server. Methods Mol. Biol..

[bib55] Woodruff M.C., Kim E.H., Luo W., Pulendran B. (2018). B Cell Competition for Restricted T Cell Help Suppresses Rare-Epitope Responses. Cell Rep..

[bib56] Yoshida N., Nussenzweig R.S., Potocnjak P., Nussenzweig V., Aikawa M. (1980). Hybridoma produces protective antibodies directed against the sporozoite stage of malaria parasite. Science.

[bib57] Zavala F., Cochrane A.H., Nardin E.H., Nussenzweig R.S., Nussenzweig V. (1983). Circumsporozoite proteins of malaria parasites contain a single immunodominant region with two or more identical epitopes. J. Exp. Med..

[bib58] Zavala F., Tam J.P., Hollingdale M.R., Cochrane A.H., Quakyi I., Nussenzweig R.S., Nussenzweig V. (1985). Rationale for development of a synthetic vaccine against Plasmodium falciparum malaria. Science.

