# Supplementary material for: Avid binding by B cells to the Plasmodium circumsporozoite protein repeat suppresses responses to protective subdominant epitopes
Source: Cell Rep. 2021 Apr 13;35(2):108996. doi: 10.1016/j.celrep.2021.108996 (PMC8052187; doi:10.1016/j.celrep.2021.108996)
Supplement: Document S1. Figures S1–S6 [file mmc1.pdf]

Cell Reports, Volume *35*

Supplemental information

Avid binding by B cells to the *Plasmodium* circumsporozoite protein repeat suppresses responses to protective subdominant epitopes

Deepyan Chatterjee, Fiona J. Lewis, Henry J. Sutton, Joe A. Kaczmarski, Xin Gao, Yeping Cai, Hayley A. McNamara, Colin J. Jackson, and Ian A. Cockburn

Figure S1

a

```
>CSP 3D7 genome sequence
  1 MMRKLAILSV SSFLFVEALF QEYQCYGSSS NTRVLNELNY DNAGTNLYNE
 51 LEMNYYGKQE NWYSLKKNSR SLGENDDGNN EDNEKLRKPK HKKLKQPADG
101 NPDPNANPNV DPNANPNVDP NANPNVDPNA NPNANPNANP NANPNANPNA
151 NPNANPNANP NANPNANPNA NPNANPNANP NANPNANPNA NPNANPNVDP
201 NANPNANPNA NPNANPNANP NANPNANPNA NPNANPNANP NANPNANPNA
251 NPNANPNANP NANPNANPNA NPNKNNQGNG QGHNMPNDPN RNVDENANAN
301 SAVKNNNNEE PSDKHIKEYL NKIQNSLSTE WSPCSVTCGN GIQVRIKPGS
351 ANKPKDELDY ANDIEKKICK MEKCSSVFNV VNSSIGLIMV LSFLFLN
```

b

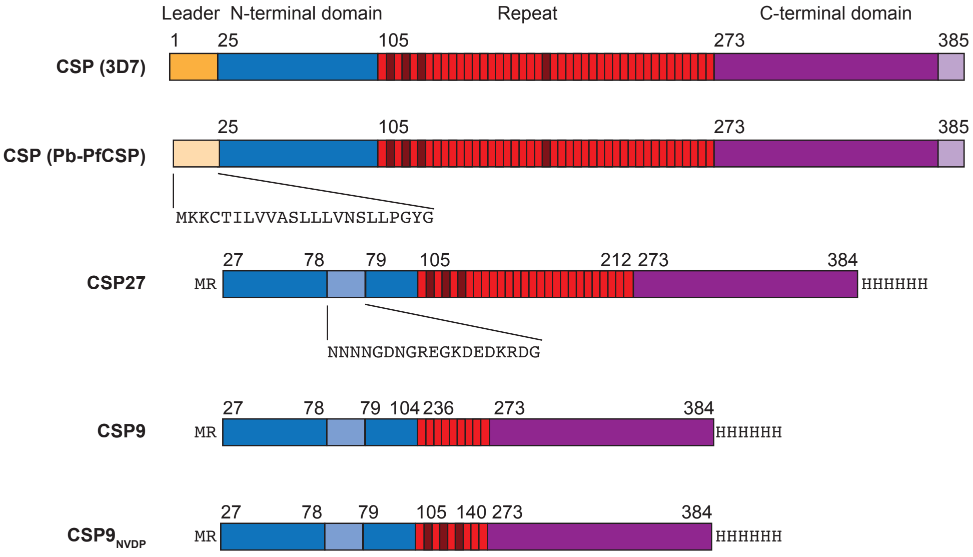

c

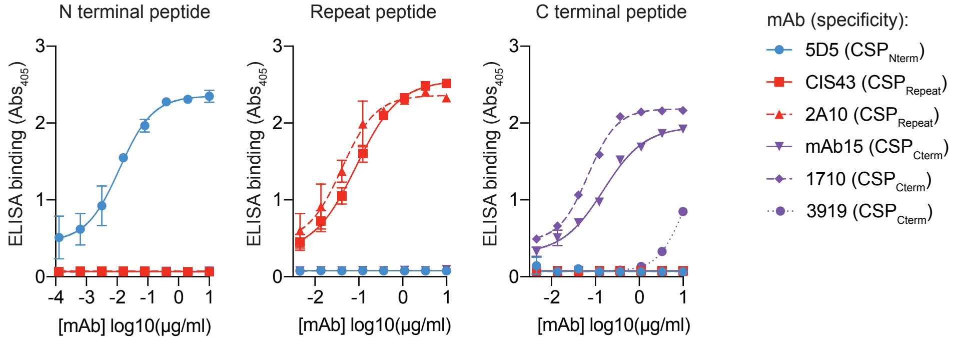

Figure S1 (related to Figure 1): Circumsporozoite protein sequences and constructs used in this study (A) Sequence of the 3D7 circumsporozoite protein, different domains are labelled with different colours: leader sequence, orange; N-terminal domain, blue; repeat, NANP – light red, NVDP – dark red; C terminal domain, purple. (B) Schematics of the different constructs used in this study, numbers refer to the 3D7 sequence, insertions/substitutions are marked as text. (C) ELISA Binding by previously described mAbs to peptides corresponding to the different domains of CSP, data representative of two independent experiments.

Figure S2

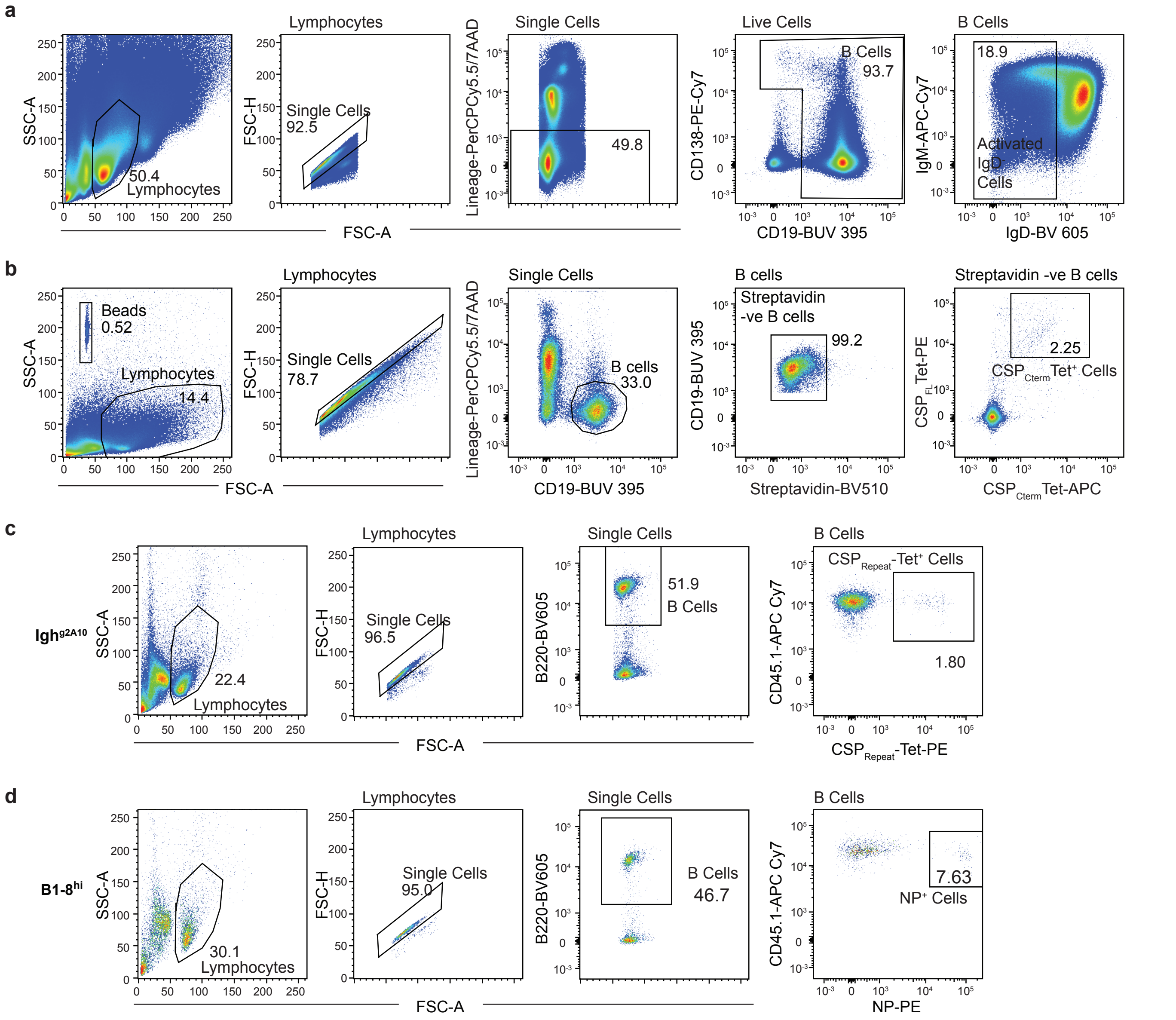

Figure 2 (related to Figures 1-5): Gating strategies for flow cytometry (A) General gating strategy applied to all flow cytometry data prior to gating antigen specific cells. Firstly, lymphocytes were gated based on their relative size and granularity (forward scatter area versus side scatter area). From the lymphocyte population, single cell events were gated on relative forward scatter area versus forward scatter height. From the single cell population, live cells were gated on PerCP Cy5.5 negative population (this is negative for 7AAD viability dye and also negative for lineage markers CD3, CD11b, CD11c and GR1). From the live cells, B cells and plasmablasts were gated as $CD19^+$ and/or $CD138^+$ cells. From the B cell population, activated cells were gated as IgD^- cells. The activated cells were then further gated for antigen specificity. Plots of further gating are provided in results figures. (B) Gating strategy to quantify CSP domain-specific precursors. Lymphocytes were gated based on their relative size and granularity (forward scatter area versus side scatter area). From the lymphocyte population, single cell events were gated on relative forward scatter area versus forward scatter height. From the single cell population, live cells were gated on PerCP Cy5.5 negative population (this is negative for 7AAD viability dye and also negative for lineage markers CD3 and GR1), we further excluded streptavidin-BV510 binding cells, and double positive cells were identified as the specific population of interest. (C) Gating strategy for quantifying Igh^{g2A10} NANP tetramer+ B Cells. Lymphocytes were gated based on their relative size and granularity (forward scatter area versus side scatter area). From the lymphocyte population, single cell events were gated on relative forward scatter area versus forward scatter height. From the single cell population, B cells were gated as $B220^+$ cells. From the B cell population, CSP_{Repeat}-tet^+ cells were gated as cells double-positive for both the tetramer and congenic marker CD45.1. (B) Gating strategy for quantifying B1-8^{Hi} NP+ B cells. The lymphocyte, single cell and B cell populations were gated as described in (A). From the B cell population, NP^+ B cells were gated as cells double-positive for both the NP marker and congenic marker CD45.1.

Figure S3

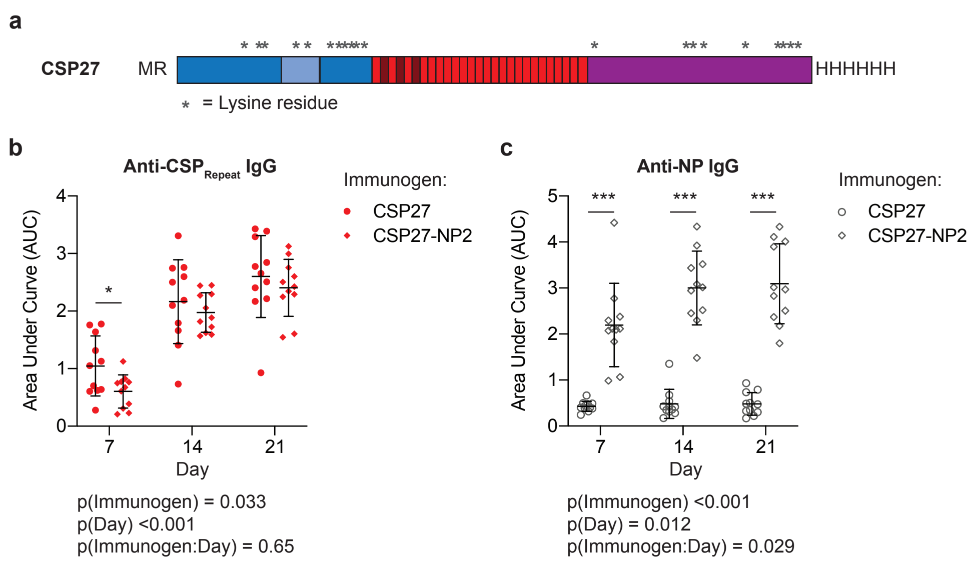

Figure S3 (related to Figures 3 and 4): CSP27-NP2 elicits both CSPRepeat and NP specific IgG responses C57BL/6 mice were immunised with CSP27-NP2 or CSP27 only control. Sera were taken 7, 14 and 21 days post-immunisation and the antibody response to each epitope measured via ELISA. (A) Schematic of CSP27 showing the locations of lysine residues, indicated by asterisks. (B) Total IgG response to CSP_{Repeat} measured via $(NANP)_9$ ELISA. (C) Total IgG response to NP measured via NP(14)BSA ELISA. Data are represented as mean ± SD pooled from three independent experiments (n≥3 mice/group/experiment); ELISA data were analysed separately for each antigen in R Studio using a mixed linear model with Immunogen (CSP27-NP2/CSP27) and day as experimental factors, experiment as a fixed factor and mouse as a random factor. Two-way ANOVA p values are listed underneath each graph, pairwise comparisons were made via Tukey post test and are represented using symbols; * p<0.05, ** p<0.01, *** p<0.001.

Figure S4

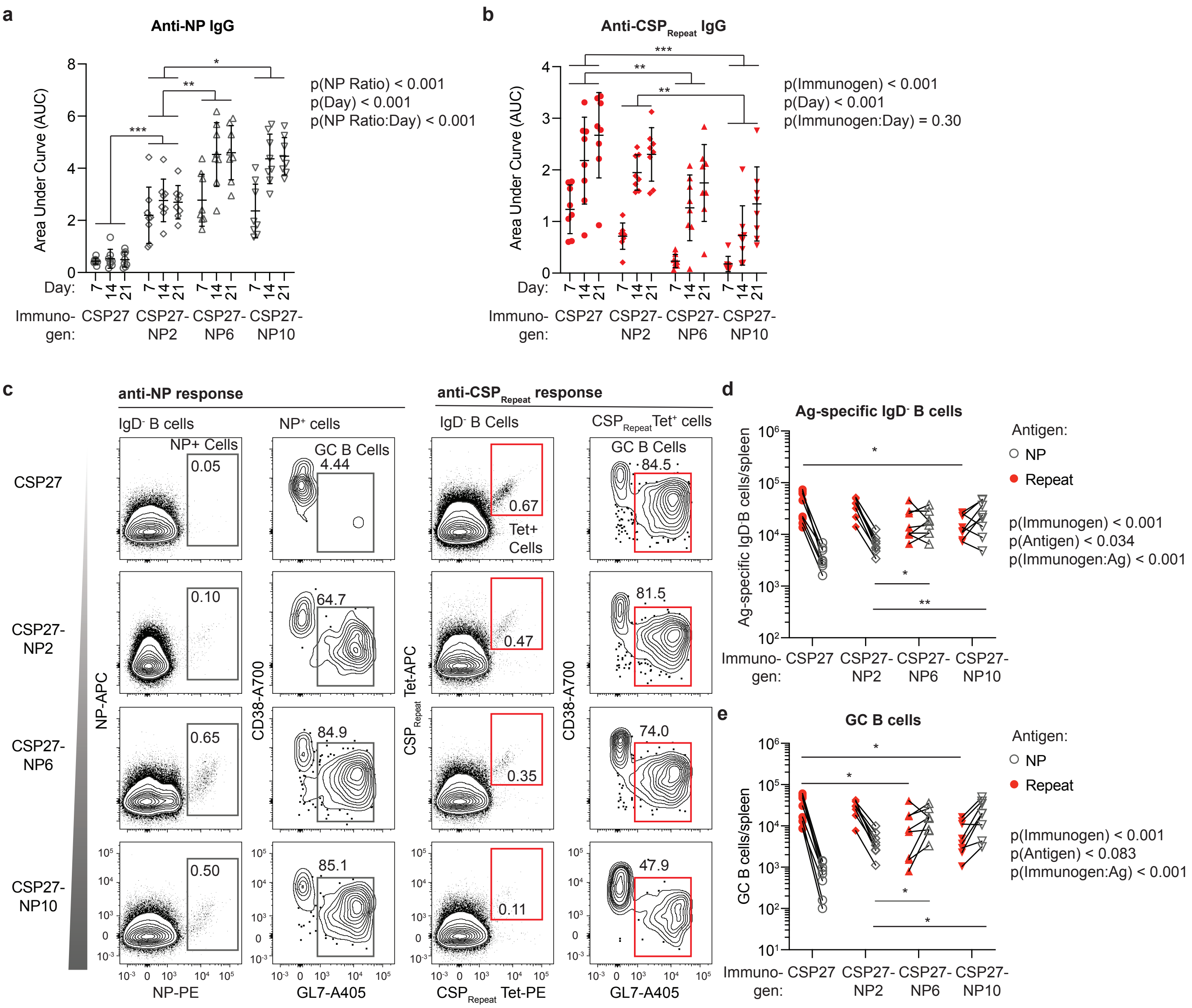

Figure S4 (related to Figure 5): Increasing the level of NP conjugation to CSP alters the immunodominance hierarchy C57BL/6 mice were immunized with either CSP27, CSP27-NP2, CSP27-NP6 or CSP27-NP10. Sera were taken on days 7, 14 and 21 and spleens analyzed 21 days post-immunization. (A) Total IgG response to NP measured via NP(14)BSA ELISA. (B) Total IgG response to CSP_{Repeat} measured via $(NANP)_9$ ELISA. (C) Representative flow cytometry plots showing gating of total IgD^- and GC B cells specific for NP or the CSP_{Repeat}; values are percentages (D) Absolute numbers of NP probe$^+$ and CSP_{Repeat} tetramer$^+$ IgD^- B cells. (E) Absolute numbers of NP probe$^+$ and CSP_{Repeat} tetramer$^+$ GC B cells. Data are represented as mean ± SD pooled from two independent experiments (n=4 mice/group/experiment); these data were analyzed via 2-way ANOVA, with experiment and mouse included in the model as fixed factors. ANOVA p values are listed below or adjacent to each graph. Pairwise comparisons were performed using a Tukey post-test with significant values are represented as symbols; * p<0.05, ** p<0.01, *** p<0.001.

Figure S5

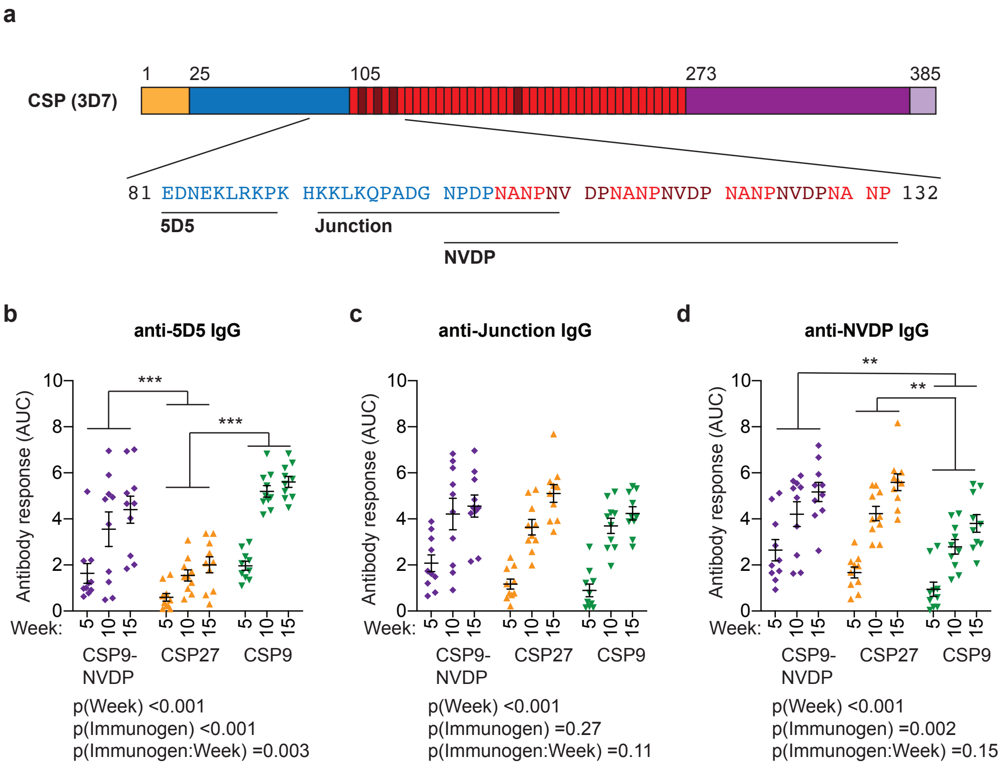

Figure S5 (related to Figure 6): Additional analysis of mice immunized with truncated CSP molecules. Sera from the mice described in Figure 4A were taken for additional ELISA analysis. (A) Sequence and location within the CSP molecule of additional peptides corresponding to potential targets of protective antibodies. (B) Overall IgG responses to the 5D5 epitope. (C) Overall IgG response to the junction between the CSP_{Nterm} and CSP_{Repeat}. (D) Overall IgG response to a peptide corresponding to the first 32 amino acids of the 3D7 CSP repeat domain including multiple NVDP repeats. Data from panels B-D was analyzed from 2 experiments with 5 mice/experiment/group analyzed via 2-way ANOVA with experiment and mouse as blocking factors, ANOVA p values are listed below or adjacent to each graph; pairwise comparisons between groups (averaged over time) were performed using a Tukey post-test and significant values are represented as symbols; * $p<0.05$, ** $p<0.01$, *** $p<0.001$.

Figure S6

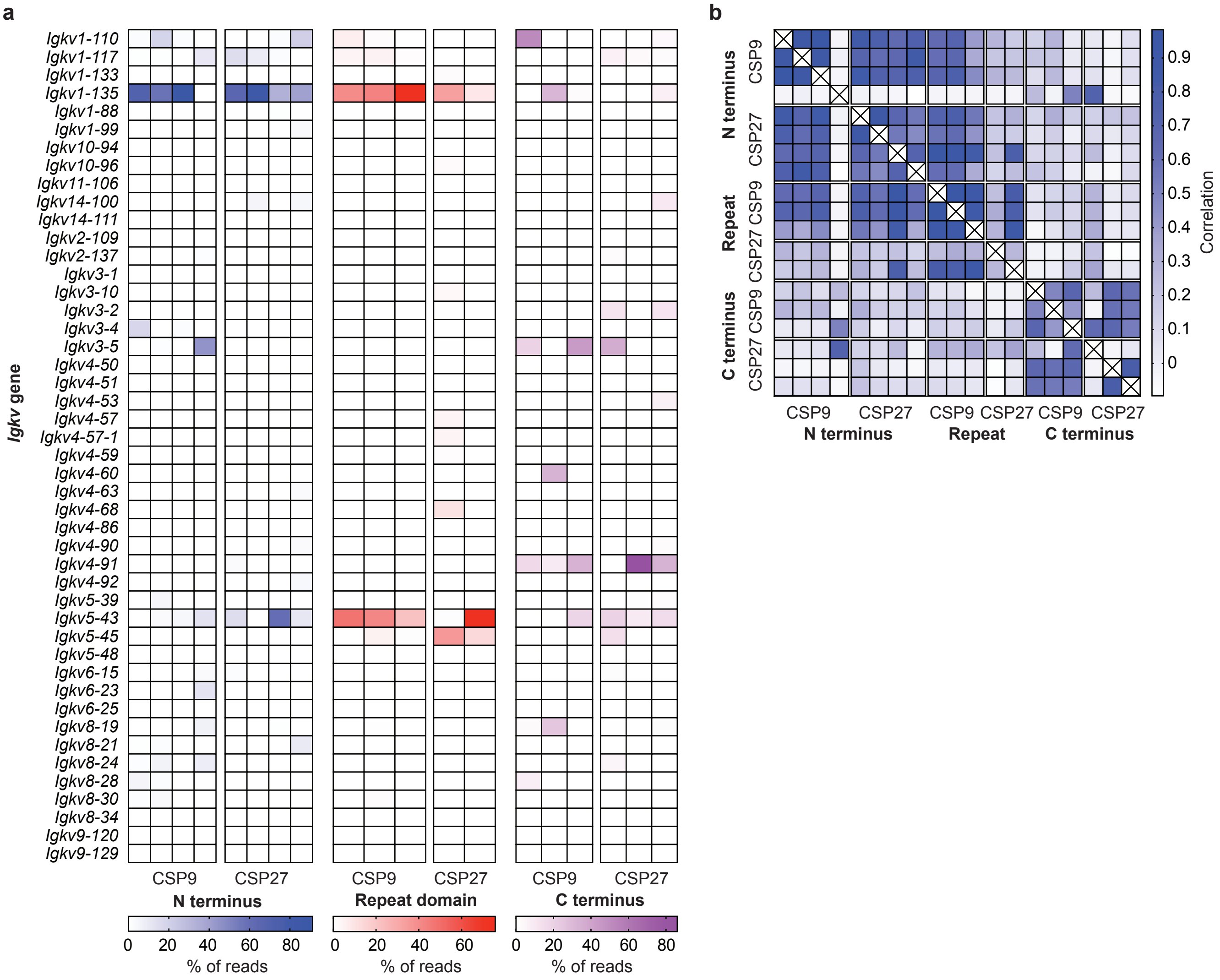

Figure S6 (related to Figure 7): *Igkv* gene usage by CSP-specific B cells in mice. CSP_{Nterm}, CSP_{Repeat} and CSP_{Cterm} specific B cells were sorted from mice immunized 10 days previously with CSP27 or CSP9 in Alum and the Igkv regions amplified with degenerate primers and sequenced. (A) *Igkv* gene usage among B cells specific for each antigen (B) correlation of Igkv gene usage for the different antigens between different mice.
